# Inhaled Micro- and Nanoplastics as Environmental Modifiers of Lung Carcinogenesis: Mechanistic Insights and Evidence Synthesis

**DOI:** 10.3390/jox16040136

**Published:** 2026-07-26

**Authors:** Chrysa Andrikopoulou, Nikolaos E. Koletsis, Vasileios Leivaditis, Francesk Mulita, Sofoklis Mitsos, Periklis Tomos, Ioannis Panagiotopoulos, Vasiliki Androutsopoulou, Marios G. Kostakis, Nikolaos S. Thomaidis, Efstratios Koletsis

**Affiliations:** 1 Department of Cardiothoracic Surgery, General University Hospital of Patras, University of Patras, 26504 Patras, Greece; chrysa661@gmail.com; 2 Department of General Surgery, General Hospital of Eastern Achaia–Unit of Aigio, 25100 Aigio, Greece; 3 Laboratory of Analytical Chemistry, Department of Chemistry, National and Kapodistrian University of Athens, Panepistimiopolis Zographou, 15772 Athens, Greece; nekoletsis@chem.uoa.gr (N.E.K.); makostak@chem.uoa.gr (M.G.K.); ntho@chem.uoa.gr (N.S.T.); 4 Department of Cardiothoracic and Vascular Surgery, Westpfalz Klinikum, 67655 Kaiserslautern, Germany; 5 Second Department of Surgery, Medical School, Democritus University of Thrace, 68100 Alexandroupolis, Greece; 6 Department of Thoracic Surgery, Attikon General Hospital, National and Kapodistrian University of Athens, 12462 Athens, Greece; sophocmit@yahoo.gr (S.M.);; 7 Department of Cardiac Surgery, Ippokrateio General Hospital of Athens, 11527 Athens, Greece; mdgiapan@yahoo.gr; 8 Department of Cardiothoracic Surgery, University Hospital of Larissa, 41110 Larissa, Greece

**Keywords:** microplastics, nanoplastics, lung cancer, inhalation exposure, pulmonary toxicity, genotoxicity

## Abstract

The exponential rise in global plastic production has resulted in the widespread environmental dissemination of micro- and nanoplastics (MNPs) across air, water, and biological systems. Inhalation of airborne MNPs represents a biologically plausible pathway of pulmonary exposure, particularly within indoor and occupational environments. Experimental evidence indicates that inhaled MNPs deposit within distal lung compartments, where their small aerodynamic diameter and surface reactivity may favor cellular uptake, oxidative stress induction, inflammatory activation, and prolonged biopersistence. Experimental studies further indicate that MNP exposure may induce DNA damage, chromosomal instability, and the dysregulation of signaling pathways involved in genomic integrity, thereby providing additional mechanistic support for their potential role in carcinogenesis. Chronic redox imbalance, macrophage dysfunction, inflammasome activation, epithelial–mesenchymal transition, and dysregulated cell adhesion collectively resemble mechanisms implicated in inflammation-associated carcinogenesis. Emerging in vitro and in vivo data further suggest that nanoplastics may function as tumor promoters or co-carcinogenic modifiers, particularly under chronic low-dose exposure or in combination with other airborne toxicants. However, human epidemiological evidence remains limited, and causality has not been established. This review synthesizes current mechanistic evidence regarding inhaled MNPs as potential modifiers of lung carcinogenesis, compares them with established inhaled carcinogens, and outlines critical research priorities necessary to clarify exposure–response relationships and clinical relevance. Current evidence supports biological plausibility rather than confirmed carcinogenic classification.

## 1. Introduction

Plastic pollution represents a major environmental challenge of the 21st century, exerting widespread and increasingly recognized impacts on ecosystems and human health [1]. The dramatic escalation of global plastic production over the past seven decades has fundamentally altered environmental exposure patterns. Fragmentation of macroplastics through mechanical abrasion, ultraviolet radiation, and oxidative degradation generates microplastics (MPs), and at smaller size ranges, nanoplastics (NPs) [1,2]. However, due to current analytical limitations, the environmental detection and quantification of airborne plastic particles remains dominated by the MP fraction, whereas NPs are less directly characterized. Increasing evidence confirms that MPs are not confined to aquatic ecosystems, but are present in indoor and outdoor air, creating continuous opportunities for inhalational exposure [3].

The respiratory tract represents a primary interface with airborne particulate exposures. Deposition patterns are governed by aerodynamic diameter, shape, density, and surface characteristics—principles well-established in the context of particulate matter, crystalline silica, and asbestos [4]. NPs, due to diffusion-dominated transport, preferentially deposit in alveolar regions, where the epithelial barrier is extremely thin and clearance mechanisms are limited [5]. Biopersistence, surface reactivity, and interaction with lung lining fluid further modulate downstream biological responses, potentially favoring sustained tissue interaction.

While early research on MNPs focused predominantly on environmental distribution and ecological impacts, more recent investigations have shifted toward the toxicological characterization of pulmonary exposure. Experimental studies report that inhaled MNPs induce oxidative stress, inflammatory signaling, mitochondrial dysfunction, endoplasmic reticulum stress, and regulated cell death pathways in lung epithelial and immune cells [6]. These responses overlap with established molecular and cellular mechanisms implicated in lung carcinogenesis, including sustained proliferative signaling, genomic instability, chronic inflammation, and remodeling of the tumor microenvironment [7].

Beyond oxidative and inflammatory responses, increasing experimental evidence suggests that MNPs may also exert direct and indirect genotoxic effects. In vitro studies have reported DNA strand breaks, chromosomal instability, oxidative DNA damage, and the dysregulation of signaling pathways involved in genome maintenance following exposure to micro- and nanoplastics, particularly in lung epithelial cells. Although these findings originate predominantly from experimental models and their relevance to human disease remains to be fully established, they provide an important mechanistic link between inhaled MNP exposure and biological processes associated with tumor initiation and progression [6,7].

Despite this growing body of evidence, existing reviews have largely addressed environmental occurrence, detection methodologies, or general organ toxicity without systematically integrating inhalation exposure, pulmonary deposition dynamics, and carcinogenic pathway convergence within a unified framework. Moreover, few analyses have contextualized MNPs alongside established inhaled carcinogens under mechanistic paradigms traditionally applied to particulate matter, silica, and asbestos [4,7]. Consequently, a structured evaluation of their potential contribution as modifiers of lung carcinogenesis remains limited.

**Figure 1:**
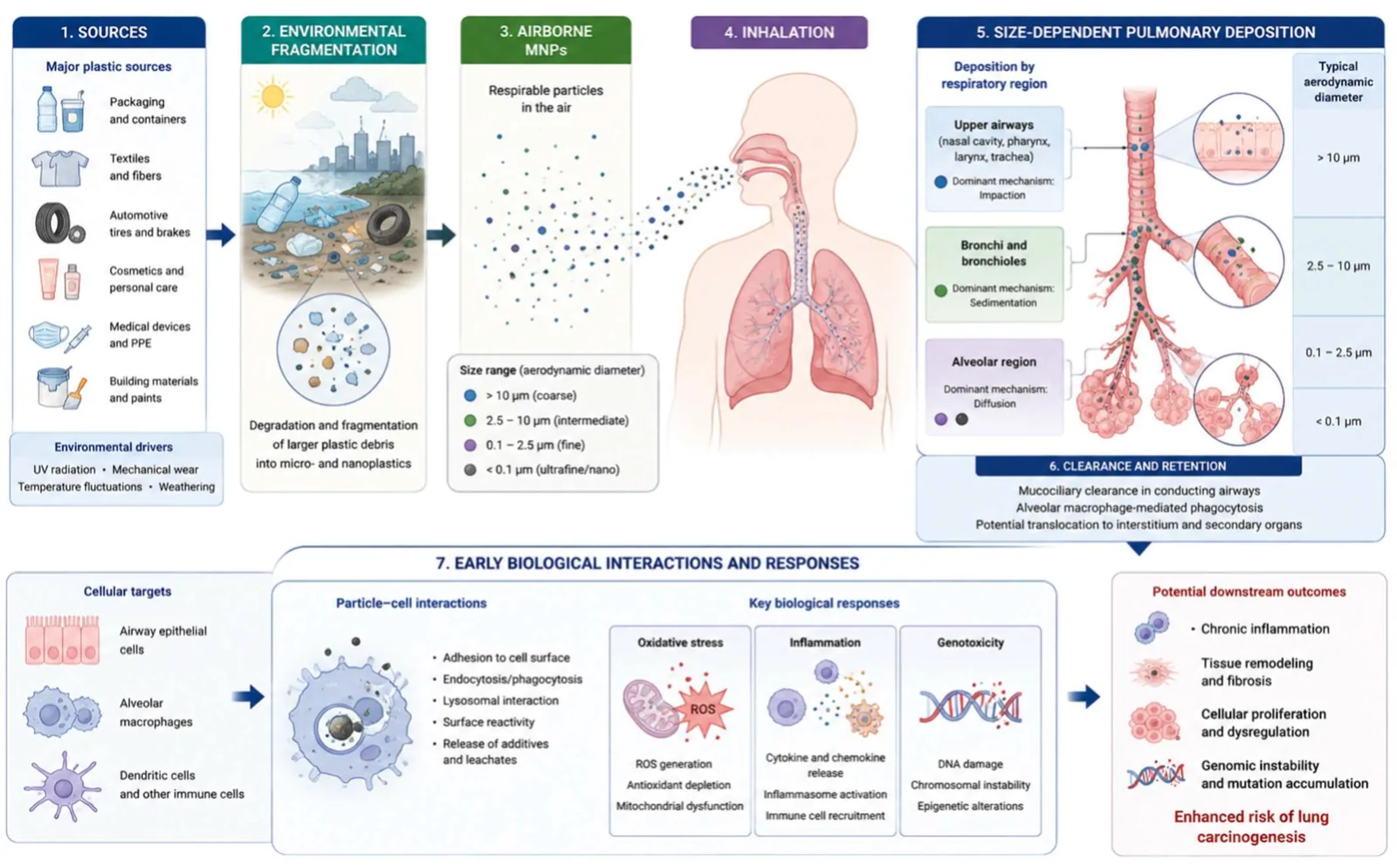
Overview of inhalation exposure pathways, pulmonary deposition, and early biological interactions of airborne micro- and nanoplastics (MNPs). Environmental fragmentation of plastic materials generates respirable particles that enter the human respiratory tract through inhalation. Deposition patterns are governed primarily by aerodynamic diameter, with larger particles depositing in the upper airways by impaction, intermediate particles in bronchiolar regions by sedimentation, and nanoscale particles reaching the alveolar compartment through diffusion-dominated transport. Following deposition, interactions with epithelial cells and alveolar macrophages may initiate oxidative stress, inflammatory signaling, and other biological responses that provide the mechanistic basis for the subsequent sections of this review.

A central consideration is whether chronic inhalational exposure to MNPs influences biological processes involved in tumor initiation, promotion, and progression. This review synthesizes current mechanistic evidence, examines deposition and retention within the respiratory tract, compares MNPs with established inhaled carcinogens, and critically evaluates pathway convergence as a basis for biological plausibility in lung cancer development. An overview of the principal inhalation exposure pathways, pulmonary deposition patterns, and early biological responses associated with airborne micro- and nanoplastics is presented in Figure 1.

## 2. Methods

### 2.1. Study Design

This study was conducted as a narrative mechanistic review aimed at synthesizing and critically evaluating current evidence regarding the potential role of inhaled MNPs in lung carcinogenesis. Given the emerging and methodologically heterogeneous nature of the field, a quantitative meta-analysis was not feasible.

### 2.2. Literature Search Strategy

A comprehensive literature search was conducted using PubMed/MEDLINE, Scopus, and Web of Science. Articles published between 2004 and January 2026 were considered. The final search was performed in January 2026. The search strategy combined controlled vocabulary (MeSH terms) and free-text keywords related to plastic particles, inhalation exposure, pulmonary toxicity, and carcinogenesis. Core search terms included “microplastics”, “nanoplastics”, “airborne plastics”, “inhalation exposure”, “lung”, “pulmonary”, “lung cancer”, “carcinogenesis”, “oxidative stress”, “inflammation”, “genotoxicity”, “epithelial–mesenchymal transition”, and “tumor microenvironment”. Boolean operators (AND/OR) were used to refine combinations of exposure- and outcome-related terms.

Because genotoxicity represents a central mechanistic pathway linking MNP exposure to carcinogenesis, additional searches specifically combining terms related to DNA damage, chromosomal instability, mutagenesis, and genomic integrity with micro- and nanoplastics were also performed to ensure comprehensive identification of relevant mechanistic studies.

### 2.3. Eligibility Criteria

Peer-reviewed articles published in English were eligible for inclusion. A total of seventy-nine (79) articles were included in the final qualitative synthesis. Eligible studies comprised:

- In vitro experimental studies involving lung epithelial cells, macrophages, airway organoids, or co-culture systems;
- In vivo animal studies assessing pulmonary exposure to MPs or NPs;
- Human biomonitoring and tissue-detection studies;
- Mechanistic, toxicological, and carcinogenesis-focused reviews providing contextual or integrative perspectives;
- Authoritative risk assessment or classification documents (e.g., IARC monographs) used for mechanistic comparison.

Studies not directly related to respiratory exposure, conference abstracts, non-peer-reviewed reports, and articles lacking mechanistic relevance to lung pathology were excluded.

### 2.4. Study Selection and Data Synthesis

Titles and abstracts were initially screened for relevance to the view scope. Full-text evaluation was subsequently performed to assess methodological quality, exposure characterization, and mechanistic relevance to lung carcinogenesis. Given the substantial heterogeneity in particle size definitions, polymer composition, surface chemistry, exposure duration, dose metrics (mass concentration, particle number, surface area), and experimental model systems, a qualitative narrative synthesis approach was adopted. The objective was not to establish causality, but to evaluate mechanistic convergence and identify knowledge gaps relevant to lung cancer risk assessment.

### 2.5. Methodological Considerations

This review acknowledges that exposure assessment methods for MNPs remain non-standardized across studies. Variability in sampling and pretreatment procedures, contamination-control strategies, analytical platforms including FTIR, Raman spectroscopy and pyrolysis-GC/MS, size-detection limits and reporting metrics complicate cross-study comparability and quantitative interpretation. In the present review, NPs are defined as the plastic particle fraction below 1 μm (<1000 nm), consistent with commonly used terminology in the nanoplastics literature. Nevertheless, some studies apply operational or method-dependent size cut-offs, contributing to heterogeneity in classification and reporting. Therefore, the qualitative synthesis emphasizes mechanistic plausibility and biological coherence rather than quantitative risk estimation.

## 3. Current Evidence on Inhaled MNPs and Lung Carcinogenesis

### 3.1. Sources and Characteristics of Airborne MNPs

Plastic production has expanded markedly since the mid-20th century, rising from less than 2 megatons annually in 1950 to more than 400 megatons today, with parallel increases in environmental dispersion and accumulation [1]. The term *microplastic* was first introduced in 2004, initiating systematic investigation into microscopic plastic debris in environmental matrices [8]. MPs are generally defined as particles <5 mm, while NPs are typically <100 nm, although some classifications extend the nanoscale range up to 1000 nm [9,10]. The rapid growth of research over the past decade reflects increasing scientific concern regarding their persistence, physicochemical complexity, and biological relevance [11].

MPs are classified as primary—manufactured at microscopic dimensions for specific industrial or consumer uses—or secondary, formed through progressive fragmentation of larger plastics under ultraviolet radiation, oxidation, hydrolysis, and mechanical abrasion [12]. Fragmentation not only reduces particle size but also modifies polymer structure, generating vast numbers of nanoscale fragments; a single gram of macroplastic may yield billions of nanoplastic particles, dramatically increasing the cumulative surface area and potential reactivity [13]. Given the continuous global input of plastic waste into marine and terrestrial systems, nanoscale plastics are likely present in extremely high numbers across environmental compartments, including the atmosphere [13].

MNPs thus represent a dynamic size continuum distributed throughout oceans, freshwater, soils, air, and biota [10,12]. Their ubiquity across environmental matrices—and documented detection in human tissues—has led some authors to characterize the current epoch as a “Plasticene”, emphasizing the planetary scale of plastic dissemination and biological integration [14]. The detection of plastic particles in human biological samples indicates that exposure is ongoing rather than hypothetical, underscoring the need for rigorous health assessment [15].

Airborne MNPs arise from diverse indoor and outdoor sources. Secondary emissions dominate and include synthetic textile fibers, tire abrasion, construction and insulation materials, paints, coatings, and industrial wear processes, all contributing to urban particulate matter [16]. Environmental analyses have identified more than 45 polymer types, with polypropylene (PP), polyethylene terephthalate (PET), and polyethylene (PE) most frequently detected [17] Additional polymer classes, including polyoxymethylene (POM), ethylene vinyl acetate (EVA), and fluorinated polyfluoroalkene polymers such as polyvinylidene fluoride (PVDF), have also been identified in environmental samples, reflecting heterogeneous sources that include food packaging, textiles, cosmetics, medical devices, and industrial applications [18]. PET and PP are strongly associated with beverage containers and synthetic fabrics, while PVC commonly derives from piping and healthcare materials [19]. Atmospheric surveys consistently report PE, PP, PS, PET, and PVC, with fluorinated polymers occasionally detected in specialized industrial settings, even in remote and high-altitude locations, supporting long-range atmospheric transport [19]. Nevertheless, methodological heterogeneity continues to constrain precise quantification and source attribution [16].

The physicochemical properties of MNPs are determined not only by the polymer itself, but also by its constituent monomers and manufacturing additives. The most frequently detected airborne polymers include polyethylene (derived from ethylene), polypropylene (propylene), polystyrene (styrene), polyvinyl chloride (vinyl chloride), and polyethylene terephthalate (terephthalic acid and ethylene glycol). Differences in polymer chemistry influence particle stability, hydrophobicity, degradation behavior, and the release of residual monomers or additives, all of which may modulate biological interactions following inhalation. Consequently, the toxicological profile of MNPs is influenced not only by particle size and morphology, but also by their underlying chemical composition.

Although less frequently detected than PE, PP, PET, or PVC, fluorinated polymers such as polytetrafluoroethylene (PTFE) and polyvinylidene fluoride (PVDF) also contribute to environmental plastic pollution and may undergo fragmentation into respirable particles. While many fluoropolymers are considered relatively chemically inert under physiological conditions, concerns have been raised regarding residual fluorinated monomers, manufacturing-related compounds, and degradation products, some of which have demonstrated genotoxic, oxidative, and carcinogenic potential in experimental studies. These observations highlight that the biological effects of MNPs may depend not only on particle size and morphology, but also on polymer-specific chemical composition and associated contaminants [17,18,19].

Representative polymer building blocks also warrant consideration because they may influence the biological behavior of MNPs. For example, bisphenol A serves as a monomer in polycarbonate plastics, whereas terephthalic acid is a principal constituent of polyethylene terephthalate (PET). Polyurethane materials, in contrast, are synthesized from diisocyanates and polyols that form urethane linkages within the polymer structure. Although these compounds are generally incorporated into stable polymer matrices, residual monomers, degradation products, and other leachable constituents may be released during manufacturing, environmental weathering, or aging, thereby potentially contributing to the toxicological profile of inhaled MNPs.

Plastics are chemically complex materials rather than inert polymer matrices alone. They contain residual monomers, production by-products, and numerous additives, with compositional complexity varying by polymer class—PVC and polyurethane being among the most chemically diverse [20]. Once in the environment, MNPs can readily adsorb exogenous contaminants, including heavy metals (Al, Cd, Cr, Cu, Ni, Pb, Zn) and persistent organic pollutants such as PAHs, PCBs, PBDEs, organochlorine pesticides (DDT and HCH), PFAS, BPA, phenolic compounds including phenol and chlorinated phenol derivatives, and emerging contaminants of drinking water, such as artificial sweeteners, which have also been reported to interact with MNPs [21]. Their high adsorption capacity reflects their large specific surface area, which increases progressively with fragmentation into smaller particles, thereby providing abundant binding sites for environmental contaminants. Experimental studies report the adsorption of perfluorocarboxylic acids (PFCAs) onto polystyrene particles, reinforcing the concept of MNPs as potential vectors for carcinogenic, mutagenic, and endocrine-disrupting substances [20,21]. This “carrier effect” is particularly relevant for inhalation exposure, where deposited particles may release sorbed toxicants locally within the respiratory tract.

Particle size is a primary determinant of environmental behavior and biological interaction. Progressive fragmentation increases particle number and total specific surface area, amplifying adsorption capacity and surface-driven interactions [22]. In environmental and biological fluids, MNPs may acquire a biomolecular “eco-corona” composed of proteins, lipids, carbohydrates, and humic substances, modifying surface reactivity, cellular recognition, and toxicological profile [16]. Although polymeric nanoplastics lack the quantum-scale electronic properties of certain inorganic nanomaterials, their reduced size may enhance diffusivity, mobility, and interfacial reactivity [22].

Reliable detection and physicochemical characterization of MNPs remain technically demanding. Because visual identification alone is prone to misclassification, accurate analysis requires the integration of complementary microscopic, spectroscopic, and analytical techniques [20,21]. Morphological characterization commonly employs optical and fluorescence microscopy, transmission electron microscopy (TEM), scanning electron microscopy (SEM), and atomic force microscopy (AFM) [21]. Chemical identification relies primarily on vibrational spectroscopic methods, with Fourier transform infrared spectroscopy (FTIR) serving as a reference technique, particularly for particles >20 μm, while Raman-based approaches—including micro-Raman and stimulated Raman spectroscopy—extend detection to smaller particle sizes and have been successfully applied to human lung tissue analysis [21]. Additional complementary techniques, including near-infrared (NIR) spectroscopy, nuclear magnetic resonance (NMR) spectroscopy, electrochemical analytical methods, laser-assisted direct infrared imaging (LDIR), optical photothermal infrared spectroscopy (O-PTIR), pyrolysis-gas chromatography-mass spectrometry (Py-GC-MS), and thermogravimetric analysis (TGA), further enhance polymer identification and chemical characterization. However, each method presents inherent methodological trade-offs, including limitations in spatial resolution, sample preparation requirements, destructive analysis, or quantitative accuracy, depending on the analytical platform employed [21,23].

Recent technological advances further refine this analytical landscape. In their 2025 review, Shao et al. emphasized progress in high-resolution vibrational spectroscopy, thermal decomposition-based mass spectrometry, and emerging imaging platforms capable of detecting smaller nanoplastic fractions with improved chemical discrimination [24]. The authors highlight the necessity of standardized sampling protocols, contamination control procedures, and harmonized reporting metrics to enable cross-study comparability. Accurate quantification is framed as a prerequisite for establishing robust exposure–response relationships in biomonitoring and exposomics research, particularly when assessing chronic disease and cancer risk [23,24].

These physicochemical and analytical considerations directly inform respiratory relevance. NPs, due to their small aerodynamic diameter and low density, can remain suspended in air for prolonged periods and deposit deeply within the lower respiratory tract [25]. Their elevated surface-to-volume ratio and enhanced cellular internalization capacity increase the probability of epithelial interaction and barrier translocation, especially at submicron dimensions [22]. Supporting this biological plausibility, in vivo experimental data indicate the preferential pulmonary accumulation of airborne NPs, accompanied by oxidative stress, inflammatory infiltration, and histopathological injury—effects reported as more pronounced than those observed with corresponding microplastic fractions [26]. In contrast, larger microparticles are more readily cleared through mucociliary mechanisms.

Taken together, the multiplicity of airborne polymer sources, chemical and additive complexity, capacity for contaminant adsorption, and surface amplification at the nanoscale, and demonstrated deep-lung deposition collectively provide a mechanistic rationale for continued investigation of inhaled MPNs in the context of pulmonary toxicity and potential lung carcinogenesis [22,24,26].

### 3.2. Inhalation Exposure and Pulmonary Deposition

Among the recognized exposure routes to MNPs, inhalation is a direct pathway to the respiratory epithelium and is increasingly recognized as relevant to pulmonary health. While ingestion remains quantitatively significant, airborne exposure—particularly in enclosed indoor environments enriched with fibers from textiles, furnishings, and consumer plastics—may facilitate repeated and often involuntary uptake [27,28]. Outdoor contributions arise from traffic-related abrasion, industrial activities, resuspended dust, marine aerosolization, and dried wastewater-derived particulates, collectively sustaining background atmospheric concentrations [9,19,29]. Owing to their low density and frequently elongated or fibrous structure, many plastic particles remain suspended for extended periods, thereby increasing the probability of respiratory entry [30].

Once inhaled, deposition within the respiratory tract follows aerodynamic principles analogous to established particulate matter dynamics. Larger inhalable particles are primarily retained in the upper airways, whereas particles in the fine and ultrafine range—comparable in dimension to PM2.5—can access the distal bronchioles and alveoli [9]. The alveolar compartment is characterized by an extensive exchange surface (~150 m^2^) and an exceptionally thin epithelial–capillary barrier, often below one micrometer, conditions that facilitate nanoparticle interaction with the air–blood interface [29]. Although comparisons with asbestos or fine particulates are sometimes invoked to illustrate shared deposition physics, current data support similarity in size-dependent penetration rather than equivalence in pathogenic potential.

Clearance mechanisms are strongly influenced by particle size and morphology. Mucociliary transport efficiently removes many larger particles toward the upper respiratory tract, where they are swallowed or expelled [9,21]. In contrast, particles reaching the alveolar region encounter macrophage-mediated phagocytosis as the primary defense. However, nanoscale particles may not be fully eliminated, either due to inefficient engulfment or intracellular processing constraints. Macrophages may also contribute to redistribution through lymphatic trafficking, while epithelial cells can internalize particles via endocytic or diffusional pathways [21]. Following epithelial crossing, systemic dissemination through blood or lymphatic circulation is biologically plausible, consistent with reports of MPs detected in multiple human tissues beyond the lung [21].

Human data further support pulmonary relevance. Zhao et al. reported the frequent detection of MPs in lung cancer specimens, with identification in a substantial proportion of analyzed samples [31]. Although such observations do not demonstrate causality, they indicate that inhaled particles can be retained within pathological lung tissue. Similar observations have also been reported in other human tissues and pathological specimens. Micro- and nanoplastics have been identified in the placenta, liver, myocardium, blood, vascular tissues, atherosclerotic plaques, thrombi, and several non-pulmonary tumor specimens, suggesting that tissue accumulation is not confined to the respiratory tract but may reflect systemic biodistribution following environmental exposure. Although these studies similarly demonstrate the presence of plastic particles rather than establish causal relationships with disease, they collectively support the concept that MNPs can persist within diverse biological microenvironments and potentially interact with tissue-specific pathological processes. In the context of lung cancer, however, pulmonary specimens remain of particular interest because inhalation represents the primary route of exposure, and the lung constitutes the first major site of particle deposition and prolonged tissue interaction [29,30,31].

Complementary experimental evidence from advanced airway organoid systems indicates that microplastic fibers may physically integrate within epithelial structures and alter tissue architecture over time, even in the absence of marked acute toxicity [32]. These findings emphasize that morphology, particularly fiber length and rigidity, may influence retention and tissue interaction.

It is important to recognize that the actual inhaled dose is difficult to quantify. Respiratory rate, age, sex, activity level, and environmental context all influence exposure, and a fraction of inhaled nanoparticles may be exhaled before deposition [33]. Consequently, current exposure models may underestimate or overestimate true alveolar burden depending on the assumptions used. Specifically, respiratory rate and physical activity increase minute ventilation, thereby increasing the number of airborne particles entering the respiratory tract and potentially enhancing distal lung deposition during prolonged exposure. Age influences airway geometry, breathing patterns, and mucociliary clearance efficiency, resulting in differences in regional particle deposition and retention between children, adults, and older individuals. Sex-related anatomical and physiological differences, including lung size, airway dimensions, and ventilatory mechanics, may likewise contribute to interindividual variability in deposited dose. In addition, environmental conditions—including airborne particle concentration, particle size distribution, humidity, and occupational or indoor exposure settings—substantially influence the inhaled particle burden and the fraction of particles reaching the alveolar compartment. Consequently, these host- and environment-related factors should be considered when interpreting toxicological studies and developing realistic exposure models, as they may significantly affect the biologically effective dose, and ultimately, the magnitude of downstream pulmonary responses. This uncertainty underscores a substantial gap between environmental measurement and biologically effective pulmonary dose [33].

Taken together, aerodynamic behavior, particle size, surface properties, and fiber morphology influence whether MNPs deposit in proximal airways, reach the alveolar region, evade clearance, or translocate systemically. The combination of deep-lung accessibility, thin barrier interface, partial clearance evasion, and documented tissue detection provides a coherent mechanistic basis for considering inhaled MNPs biologically relevant in pulmonary pathology and potentially in lung carcinogenesis.

### 3.3. Interaction of MNPs with Lung Tissue

Once deposited within the respiratory tract, MNPs encounter the pulmonary surfactant layer that coats the alveolar surface. This lipid–protein film constitutes the initial biological interface and plays a critical role in modulating particle behavior. Experimental and modeling data indicate that nanoparticle physicochemical characteristics—such as hydrophobicity and surface chemistry—determine whether particles diffuse across the surfactant layer or become incorporated into lipid-rich domains, acquiring a modified surface profile often described as a biomolecular corona [9]. Because surfactant function relies on tightly regulated lipid organization and protein composition, perturbation of this interface may compromise alveolar stability and impair gas exchange [34]. Thus, the surfactant–particle interaction represents an important early event shaping downstream cellular responses.

Following this initial contact, nanoscale particles may undergo epithelial internalization. Integrin α5β1-mediated endocytosis has been identified as a receptor-dependent pathway facilitating nanoplastic uptake in lung epithelial cells [35]. In the alveolar compartment, type II pneumocytes (ATII cells) maintain surfactant homeostasis through the tightly coordinated activity of lamellar bodies and associated endoplasmic reticulum and Golgi pathways; interference with these intracellular systems may disrupt surfactant production and recycling [34]. NPs that penetrate distal airways may be taken up by epithelial cells via endocytic processes, subsequently engaging redox-sensitive signaling cascades. Reported consequences include increased reactive oxygen species (ROS) generation, inflammatory mediator release, epithelial barrier alteration, and programmed cell death activation [9]. In contrast, larger particles are more commonly retained in proximal airways, where ciliated and mucus-producing epithelial cells contribute to mechanical clearance [34].

Beyond oxidative stress and inflammatory activation, increasing experimental evidence indicates that MNPs may also exert direct and indirect genotoxic effects. Excessive ROS generation can induce oxidative DNA lesions, single- and double-strand DNA breaks, chromosomal aberrations, micronucleus formation, and impaired DNA repair, thereby promoting genomic instability. Although these effects appear to be mediated predominantly through oxidative and inflammatory mechanisms, several experimental studies have also suggested that nanoscale plastics may interact directly with intracellular macromolecules, further contributing to genomic damage. Collectively, these genotoxic alterations represent a biologically plausible mechanism linking persistent MNP exposure to malignant transformation [34,35].

Particle geometry and airflow dynamics further influence deposition and subsequent tissue interaction. Experimental modeling suggests that cylindrical microplastics in the micrometer range may exhibit higher deposition efficiency under lower airflow conditions compared with certain smaller cylindrical forms, while spherical and tetrahedral particles may display comparable deposition patterns at similar flow rates [36]. Moreover, reduced airflow rates appear to favor overall particle retention across size classes [35,36]. These findings underscore that not only size but also shape, as well as respiratory mechanics, modulate local particle burden and thus potential tissue exposure.

Macrophage engagement constitutes a central immunological response following deposition. Resident alveolar macrophages are responsible for phagocytic clearance; however, sustained exposure or biopersistent particles may exceed the clearance capacity. The efficiency of pulmonary clearance is influenced by both cumulative exposure duration and the deposited particle burden. Under low-level or intermittent exposure, alveolar macrophages are generally capable of engulfing and removing a substantial proportion of deposited particles through mucociliary transport and lymphatic drainage. However, repeated or prolonged exposure may progressively increase the retained particle load, particularly for poorly degradable nanoplastics that persist within the alveolar compartment. As intracellular particle accumulation increases, macrophage phagocytic capacity may become compromised, resulting in delayed clearance, prolonged particle residence, and sustained interaction with epithelial and immune cells. Experimental studies further suggest that higher particle doses are associated with greater oxidative stress, inflammatory activation, and macrophage dysfunction, indicating that both exposure duration and cumulative deposited dose influence the efficiency of pulmonary clearance and the magnitude of subsequent toxicological responses. Polystyrene and polyethylene MNPs have been reported to impair macrophage metabolic activity, increase ROS production, and stimulate the release of pro-inflammatory cytokines [37,38,39]. Such metabolic and functional disruption of macrophages may weaken innate immune defense while simultaneously promoting a pro-inflammatory microenvironment. Mechanistically, activated alveolar macrophages respond to persistent particle exposure by releasing a broad spectrum of pro-inflammatory mediators, including tumor necrosis factor-α (TNF-α), interleukin (IL)-1β, IL-6, and various chemokines that recruit additional inflammatory cells to the site of particle deposition. Concurrent activation of redox-sensitive signaling pathways, particularly nuclear factor-κB (NF-κB) and the NLRP3 inflammasome, further amplifies cytokine production and perpetuates inflammatory signaling. Under conditions of persistent particle retention and incomplete clearance, this self-sustaining inflammatory milieu may promote epithelial injury, aberrant tissue repair, extracellular matrix remodeling, and fibrosis, thereby establishing a microenvironment that favors tumour-promoting processes. Persistent macrophage activation is therefore mechanistically associated with chronic inflammatory signaling and progressive tissue remodeling [9,34].

Biopersistence may amplify these effects. Unlike the conducting airways, the alveolar region lacks efficient mucociliary removal, increasing the likelihood of long-term retention for small or poorly degradable particles [34]. In their comprehensive analysis of nanoparticle biokinetics, Geiser and Kreyling emphasize that diffusion-driven transport promotes the efficient deposition of nanosized particles in the alveolar region, where the extremely thin air–blood barrier permits partial translocation into systemic circulation [40]. Importantly, macrophage-mediated clearance appears incomplete, and intracellular persistence of nanoparticles has been documented, resulting in slow elimination kinetics. Under repeated exposure conditions, such retention may increase the cumulative tissue interaction time, thereby enhancing the plausibility of chronic pulmonary effects [40]. Surface modifications, including protein and lipid corona formation, may further alter intracellular trafficking and degradation pathways, potentially prolonging cellular stress responses [9].

Beyond local lung effects, emerging in vivo data suggest that NP exposure can influence systemic immune homeostasis through microbiome-mediated mechanisms. In the study by Kaluç et al., chronic murine exposure to NPs altered both intestinal and pulmonary microbiota composition and was associated with immune modulation and low-grade inflammatory signaling, supporting the concept of bidirectional communication along the gut–lung axis [41]. Such findings indicate that pulmonary particle retention may not only drive local oxidative and inflammatory stress, but also interact with systemic immune–metabolic networks.

Interpretation of experimental animal studies should nevertheless consider the substantial heterogeneity of current exposure paradigms. Existing murine models differ considerably with respect to administered particle dose, particle size, and polymer composition, exposure route (e.g., inhalation, intratracheal instillation, or oropharyngeal aspiration), exposure frequency, and overall study duration. While acute high-dose exposure models are valuable for elucidating biological mechanisms, they may not accurately reflect the chronic, low-dose environmental exposures experienced by humans. Conversely, longer-term exposure studies more closely approximate real-world conditions but remain limited in number and employ widely variable experimental protocols. This methodological diversity complicates direct comparisons between studies and underscores the need for standardized, environmentally relevant exposure models to improve the translational relevance of experimental findings [40,41].

Collectively, interactions at the surfactant interface, epithelial barrier, and macrophage level—modulated by particle size, shape, surface chemistry, and biokinetic behavior—form a mechanistic basis for nanoplastic-induced pulmonary toxicity. Through oxidative stress induction, inflammatory amplification, incomplete clearance, and potential systemic translocation, inhaled MNPs establish biological conditions that may predispose lung tissue to chronic injury and tumor-promoting processes [40].

**Figure 2:**
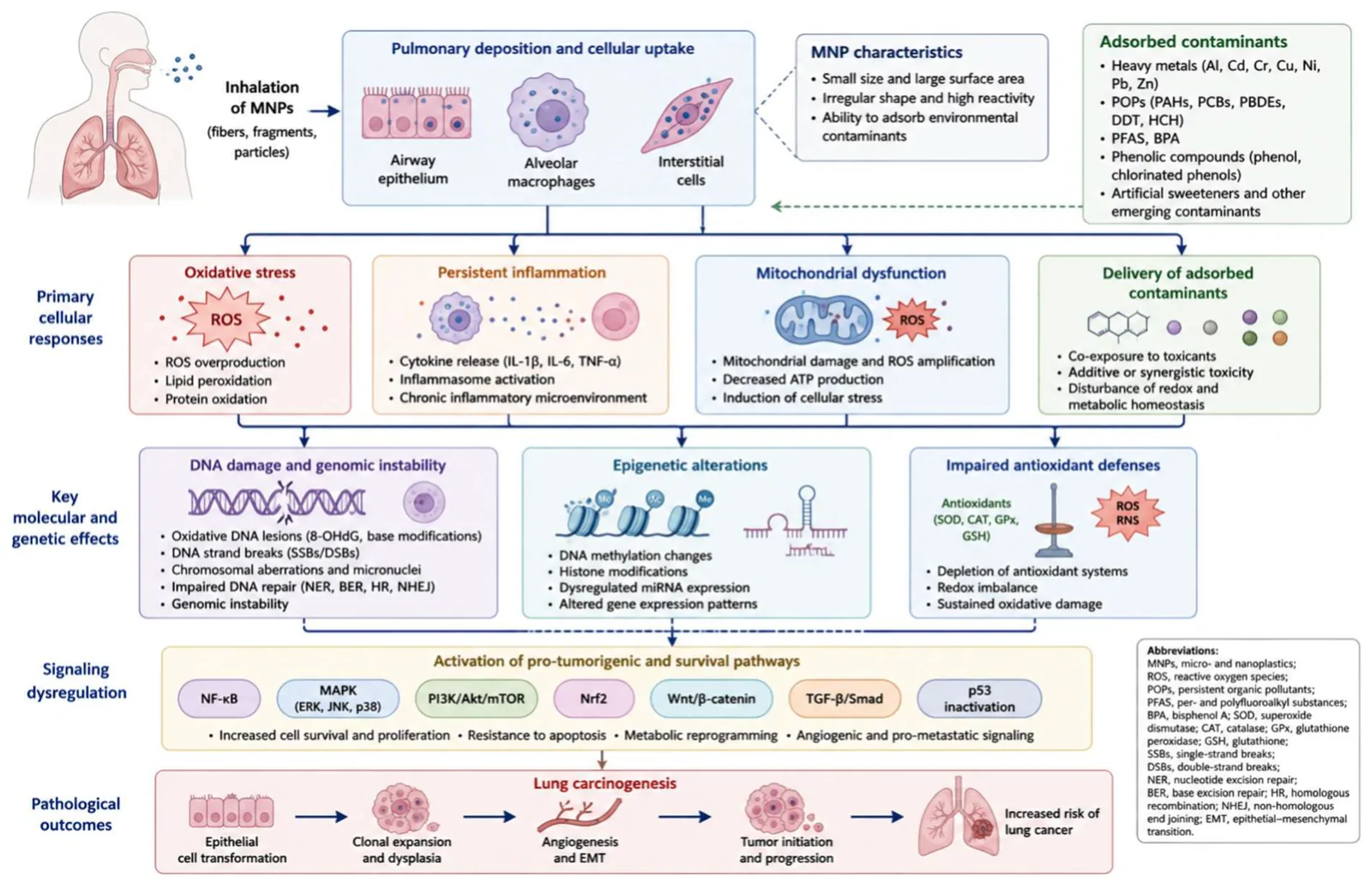
Proposed mechanisms linking inhaled micro- and nanoplastics (MNPs) to lung carcinogenesis. Following inhalation, MNPs deposit within the respiratory tract and are internalized by airway epithelial cells, alveolar macrophages, and interstitial cells. Their physicochemical characteristics and ability to adsorb environmental contaminants amplify cellular toxicity, promoting oxidative stress, persistent inflammation, mitochondrial dysfunction, and the disruption of redox homeostasis. These processes contribute to DNA damage and genomic instability—including oxidative DNA lesions, DNA strand breaks, chromosomal aberrations, and impaired DNA repair—as well as epigenetic alterations affecting gene expression. The resulting dysregulation of key signaling pathways involved in cell survival, proliferation, apoptosis, and angiogenesis ultimately promotes epithelial transformation, tumor initiation, and lung cancer progression. The figure summarizes the current mechanistic evidence derived predominantly from experimental studies.

Persistent oxidative stress and chronic inflammation are unlikely to act in isolation but rather converge with genotoxic and epigenetic mechanisms that reinforce one another during prolonged particle exposure. Oxidative DNA damage, impaired genomic maintenance, and alterations in DNA methylation, histone modifications, and non-coding RNA expression may collectively disrupt normal cellular homeostasis, promoting aberrant gene expression, resistance to apoptosis, and progressive acquisition of tumor-promoting phenotypes. The complex molecular mechanisms through which inhaled micro- and nanoplastics may contribute to lung carcinogenesis—including oxidative stress, chronic inflammation, mitochondrial dysfunction, DNA damage and genomic instability, epigenetic alterations, and dysregulation of oncogenic signaling pathways—are summarized schematically in Figure 2.

## 4. Interplay Between Oxidative Stress and Inflammatory Responses

Chronic oxidative stress and sustained inflammatory signaling represent central biological axes through which inhaled MNPs may influence pulmonary carcinogenic trajectories [42,43,44]. Rather than reintroducing deposition dynamics or clearance mechanisms discussed previously, this section focuses specifically on downstream redox–immune perturbations.

Experimental evidence indicates that polystyrene-MNPs can interact with pulmonary surfactant components and indirectly promote reactive oxygen species (ROS) generation through the disruption of cellular redox homeostasis rather than by acting as direct redox catalysts [45,46]. Proposed mechanisms include mitochondrial dysfunction, activation of NADPH oxidases, lysosomal stress following particle internalization, and inflammatory-cell activation, all of which contribute to increased intracellular ROS production. In addition, adsorbed environmental contaminants or plastic-associated additives present on particle surfaces may further amplify oxidative stress responses under certain exposure conditions [46,47,48,49]. Sustained ROS amplification may disrupt intracellular redox homeostasis and activate stress-responsive signaling pathways implicated in genomic instability and tissue remodeling [19,50].

Oxidative stress and inflammation are closely interconnected biological processes rather than independent mechanisms. Excessive ROS production can activate multiple redox-sensitive signaling pathways, including nuclear factor-κB (NF-κB), mitogen-activated protein kinase (MAPK), and the NLRP3 inflammasome, resulting in the increased expression of pro-inflammatory cytokines such as TNF-α, IL-1β, and IL-6. In turn, activated inflammatory cells—including alveolar macrophages and recruited neutrophils—generate additional ROS through NADPH oxidase activity and other oxidative pathways, thereby further amplifying oxidative stress. This reciprocal interaction establishes a self-perpetuating cycle of oxidative injury and chronic inflammation that contributes to persistent epithelial damage, tissue remodeling, and the development of a tumor-promoting pulmonary microenvironment.

Beyond sustaining inflammatory signaling, persistent oxidative stress may also promote both genotoxic and epigenetic alterations that contribute to carcinogenesis. Excessive ROS can induce oxidative DNA lesions, single- and double-strand DNA breaks, chromosomal instability, and impaired DNA repair, thereby facilitating the accumulation of genomic alterations. At the epigenetic level, experimental studies further suggest that MNP exposure may alter DNA methylation patterns, histone modifications, and non-coding RNA expression, leading to the dysregulated transcription of genes involved in oxidative stress responses, inflammation, cell-cycle regulation, apoptosis, and tumor suppression. Rather than acting independently, these genetic and epigenetic alterations appear to reinforce one another, promoting persistent cellular dysfunction and increasing susceptibility to malignant transformation.

**Figure 3:**
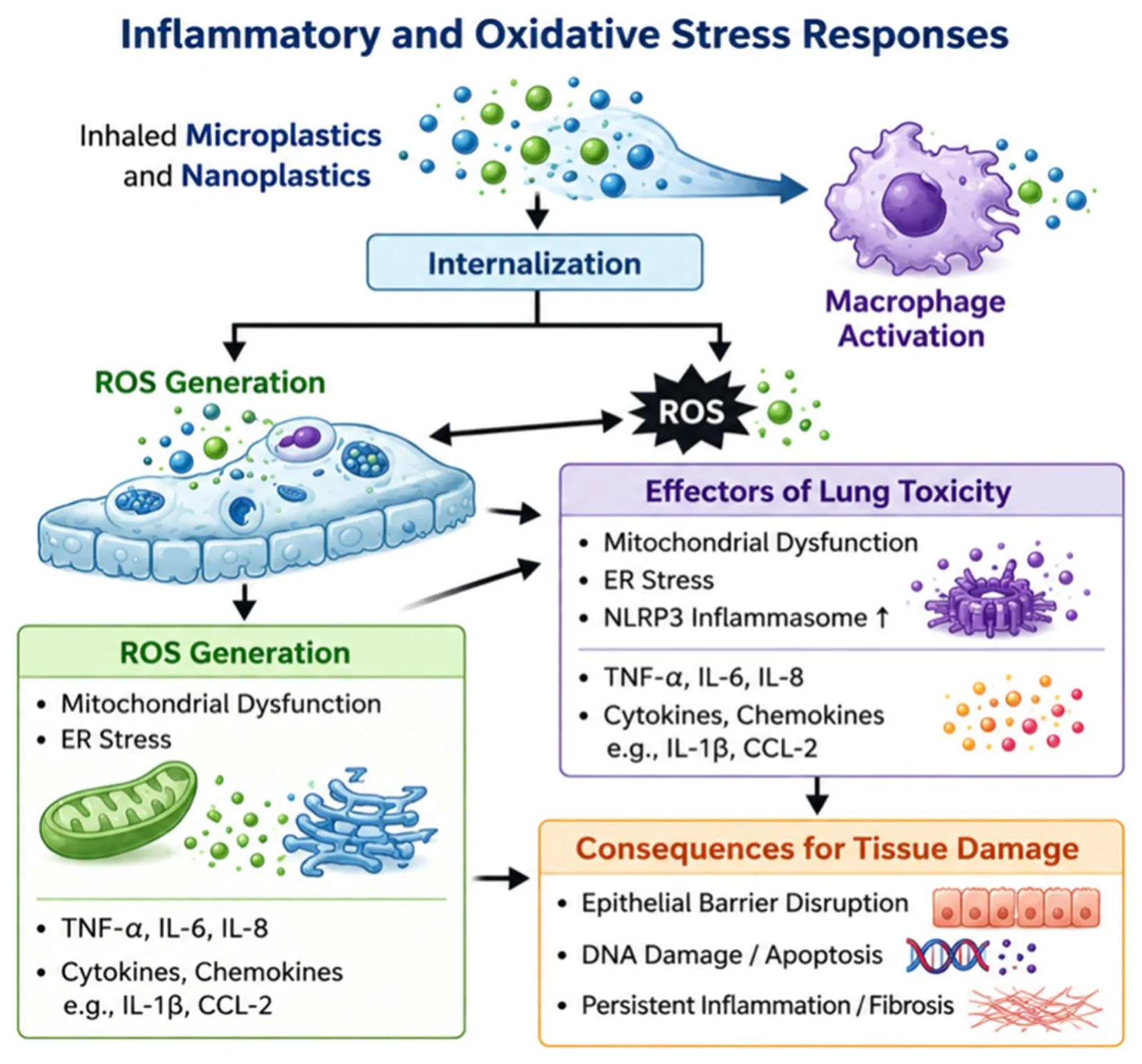
Primary biological responses associated with inhaled MNPs in pulmonary cells. Experimental studies demonstrate increased reactive oxygen species (ROS) production, mitochondrial dysfunction, endoplasmic reticulum stress, and the activation of inflammatory signaling pathways. Sustained redox imbalance and chronic inflammatory activation represent plausible mechanistic links to carcinogenesis-associated processes.

Inflammatory amplification represents a downstream and mutually reinforcing consequence of oxidative stress. Upregulation of pro-inflammatory cytokines and chemokines has been observed in pulmonary exposure models, with particular relevance attributed to NLRP3 inflammasome activation and IL-1β signaling [46,47]. Persistent inflammasome engagement has been associated with chronic immune activation and structural remodeling processes (Figure 3).

Macrophage responses further reinforce this inflammatory environment. Exposure to combustion-derived and polymer-derived nanoplastics has been reported to impair macrophage metabolic function, enhance oxidative stress, alter lipid handling, and dysregulate cytokine secretion [48,49]. These alterations may simultaneously weaken immune surveillance and perpetuate unresolved inflammation.

At the epithelial level, nanoplastic exposure has been shown to increase ROS production, promote EMT-associated marker expression, and enhance migratory behavior in both non-malignant and malignant lung cells [19,50,51,52]. Co-exposure models demonstrate synergistic inflammatory amplification in the presence of ozone [51], while ROS-dependent ER stress and ferroptotic signaling have been documented in vivo [53]. Air–liquid interface systems suggest that inflammatory activation occurs under physiologically relevant exposure conditions [54].

Collectively, these findings support a mechanistic framework in which oxidative imbalance, chronic inflammatory signaling, genotoxicity, epigenetic dysregulation, macrophage metabolic reprogramming, and epithelial plasticity converge on pathways classically associated with inflammation-driven carcinogenesis [42,43,44,54,55,56].

## 5. Genotoxic and Epigenetic Signatures in MNP-Induced Lung Carcinogenesis

Building on the inflammatory–redox framework described above, this section focuses specifically on the genomic and oncogenic consequences of inhaled MNP exposure rather than reiterating upstream oxidative mechanisms [57,58,59,60]. Increasing evidence suggests that MNPs influence multiple hallmarks of carcinogenesis through interconnected processes involving genomic instability, dysregulated intracellular signaling, mitochondrial dysfunction, epigenetic remodeling, and persistent inflammatory activation. Although much of the available evidence originates from experimental in vitro and animal models, the remarkable convergence of molecular findings across different exposure systems supports the biological plausibility of MNPs as environmental modifiers of lung carcinogenesis [61,62].

In vitro studies have consistently demonstrated that polystyrene nanoplastics induce DNA strand breaks, chromosomal instability, and the activation of proliferative signaling cascades in non-malignant lung epithelial cells [19,60,63]. Importantly, non-malignant epithelial cells appear more susceptible to early transformation-associated molecular perturbations than established lung cancer cell lines [19,64], suggesting that MNP exposure may preferentially influence the initiation rather than the progression of malignant disease. Recent investigations further indicate that MNP-induced genotoxicity extends beyond simple DNA strand disruption and includes oxidative DNA lesions, micronucleus formation, replication stress, chromosomal aberrations, and impaired DNA damage response pathways [65,66]. Excessive production of reactive oxygen species, mitochondrial dysfunction, and chronic inflammatory signaling collectively contribute to these alterations by overwhelming endogenous DNA repair mechanisms and promoting progressive genomic instability, one of the defining hallmarks of carcinogenesis [62,65].

Accumulating evidence also indicates that MNP-induced genotoxicity is closely integrated with alterations in multiple oncogenic signaling pathways. Network toxicology analyses have demonstrated significant enrichment of MAPK and PI3K/Akt signaling (55), while broader mechanistic studies suggest the simultaneous dysregulation of several additional pathways implicated in tumor initiation and progression, including Wnt/β-catenin, p53, JAK/STAT, Nrf2/Keap1, and TGF-β/Smad signaling [62,67]. These signaling networks regulate fundamental cellular processes such as proliferation, apoptosis, oxidative stress responses, epithelial–mesenchymal transition, extracellular matrix remodeling, and immune regulation. Rather than acting independently, these pathways exhibit extensive molecular crosstalk, allowing persistent oxidative stress and inflammatory signaling to reinforce cellular reprogramming and promote a tumor-permissive microenvironment [61,62,67].

Beyond direct DNA damage, recent studies suggest that chronic MNP exposure may influence genome stability through epigenetic mechanisms. Experimental evidence indicates alterations in DNA methylation patterns, histone modifications, and non-coding RNA expression following MNP exposure, thereby modifying the transcription of genes involved in antioxidant defense, inflammatory signaling, DNA repair, cell-cycle regulation, and apoptosis [62,68]. These epigenetic alterations may persist even after the initial oxidative insult has resolved, potentially contributing to sustained cellular dysfunction and increasing susceptibility to malignant transformation. Importantly, genetic and epigenetic mechanisms appear to reinforce one another, with oxidative DNA damage influencing chromatin organization and DNA repair efficiency, while epigenetic dysregulation may further impair genomic maintenance and facilitate mutation accumulation [62,68,69]. Together, these data support biological coherence between MNP-induced cellular stress pathways and mechanisms implicated in tumor initiation and progression, while recognizing that epidemiological confirmation remains pending [12,26].

Experimental animal studies further support the biological plausibility of MNP-associated carcinogenesis. In vivo murine models have demonstrated altered expression of stress- and tissue remodeling-associated genes, including *Egr1, Lamb3*, and *Scel*, together with the activation of epithelial–mesenchymal transition (EMT), mitochondrial dysfunction, and persistent oxidative stress following pulmonary exposure [56]. Chronic inhalation models have additionally reported progressive inflammatory infiltration, extracellular matrix remodeling, pulmonary fibrosis, and tumor formation in susceptible rodent strains, although direct evidence specifically implicating nanoplastics as independent carcinogens remains limited [56]. Importantly, increasing evidence suggests that prolonged particle retention and repeated low-dose exposure, rather than acute cytotoxicity alone, are likely to represent the principal drivers of carcinogenesis-related responses. Long-term exposure appears to sustain oxidative stress, impair mitochondrial bioenergetics, and perpetuate inflammatory signaling, thereby creating a biological environment conducive to malignant transformation [70,71].

**Figure 4:**
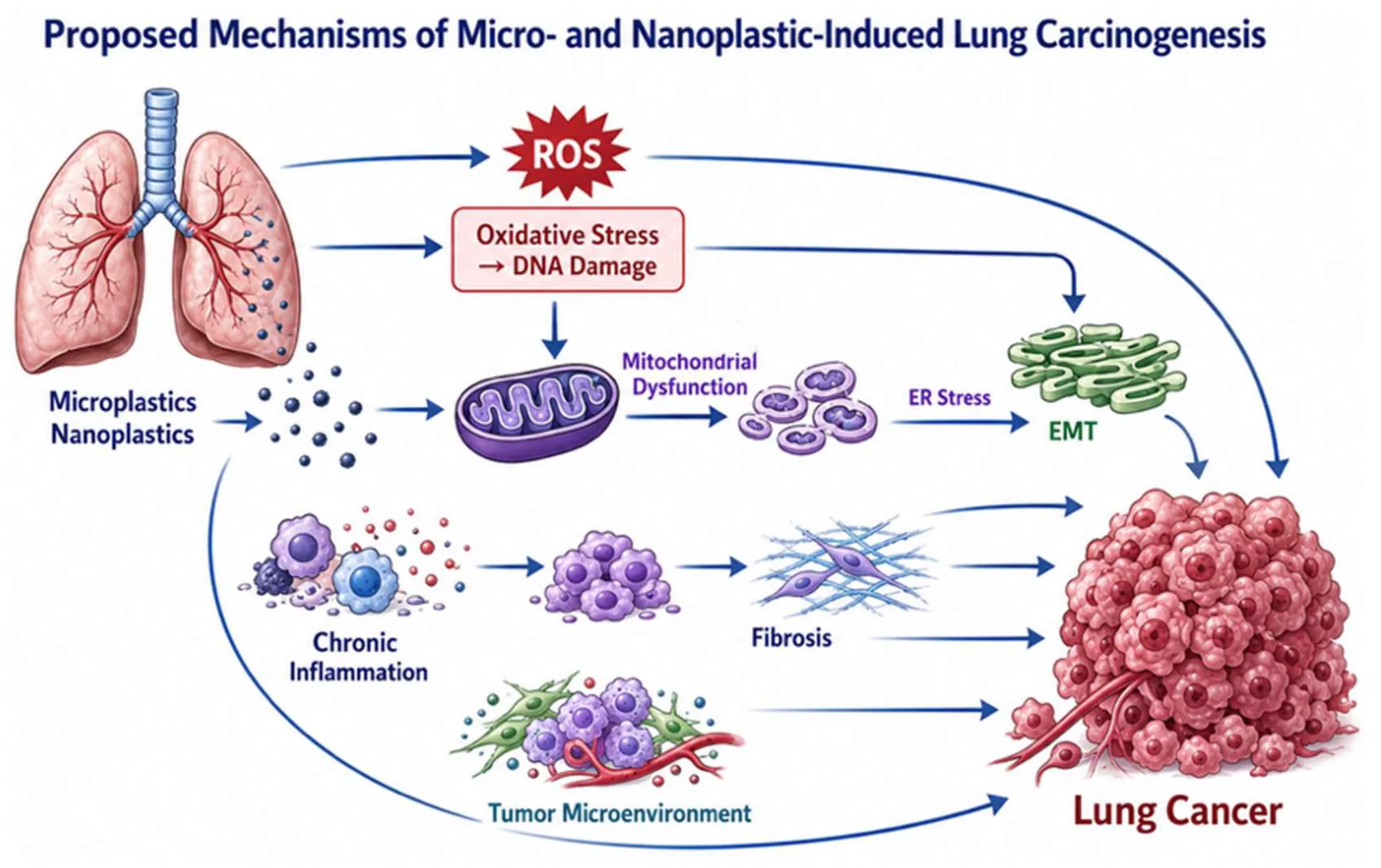
Conceptual model summarizing experimentally reported pathways linking inhaled MNPs to carcinogenesis-related processes. Following pulmonary deposition, MNPs induce reactive oxygen species (ROS) generation and oxidative DNA damage, leading to mitochondrial dysfunction and endoplasmic reticulum (ER) stress. Persistent redox imbalance promotes epithelial–mesenchymal transition (EMT), chronic inflammation, fibrosis, and tumor microenvironment remodeling. The convergence of these pathways supports mechanistic plausibility for a contributory role of MNP exposure in lung cancer development. This schematic represents an integrative model based on experimental and mechanistic evidence rather than confirmed causal classification.

Mitochondria have emerged as central regulators of this process. Beyond their essential role in cellular energy production, mitochondria coordinate apoptosis, intracellular calcium homeostasis, and redox signaling. Experimental studies indicate that MNP exposure disrupts mitochondrial membrane potential, impairs oxidative phosphorylation, alters ATP production, and enhances mitochondrial ROS generation, ultimately amplifying oxidative DNA damage and inflammatory responses [53,70]. Simultaneously, endoplasmic reticulum stress, ferroptotic signaling, defective autophagy, and altered mitophagy have all been described following nanoplastic exposure, suggesting that organelle dysfunction represents an important mechanistic link between chronic particle retention and carcinogenesis [71,72]. These findings further support the concept that persistent MNP exposure induces integrated disturbances in cellular homeostasis rather than isolated toxicological effects (Figure 4).

Tumor-associated signaling mediators—including TNF-α, NF-κB, and integrin α5β1—have been reported to be upregulated following micro- and nanoplastic exposure [57], linking particle interaction to adhesion, migration, and invasion pathways relevant to tumor progression. More recent investigations indicate that MNPs may additionally reshape the tumor microenvironment by modulating immune-cell recruitment, extracellular matrix organization, angiogenesis, stromal cell activation, and metabolic reprogramming [62,73]. Chronic activation of inflammatory and profibrotic signaling pathways may therefore promote a permissive microenvironment that facilitates epithelial plasticity, tumor cell survival, and progressive disease evolution. Although these observations remain largely experimental, they further support the hypothesis that MNPs may function as environmental modifiers of tumor biology rather than acting solely as direct mutagens [73,74].

The carrier effect may provide an additional mechanism amplifying carcinogenic potential. Owing to their large specific surface area and pronounced adsorption capacity, MNPs readily bind polycyclic aromatic hydrocarbons, heavy metals, persistent organic pollutants, endocrine-disrupting compounds, and other environmentally relevant toxicants [12,16]. Consequently, inhaled particles may act as vectors that locally concentrate these contaminants within pulmonary tissues, thereby enhancing oxidative stress, inflammatory signaling, and DNA damage beyond the intrinsic biological effects of the plastic particles themselves. Rather than representing independent mechanisms, particle toxicity and contaminant transport are likely to interact synergistically, producing cumulative biological effects that exceed those attributable to either component alone [61,62,75].

Despite these mechanistic advances, several important limitations remain. Considerable heterogeneity exists among experimental studies with respect to polymer composition, particle size, morphology, surface functionalization, administered dose, exposure route, and exposure duration, complicating direct comparisons and limiting extrapolation to environmentally relevant human exposure scenarios. Moreover, many experimental investigations employ commercially manufactured spherical polystyrene particles at concentrations substantially exceeding those currently encountered under typical environmental conditions. Consequently, although these models have been invaluable for elucidating biological mechanisms, they may not fully reproduce the physicochemical complexity or chronic low-dose exposures characteristic of real-world airborne MNPs [61,76].

Collectively, current evidence supports a biologically coherent framework in which inhaled MNPs promote oxidative stress, chronic inflammation, mitochondrial dysfunction, genomic instability, epigenetic dysregulation, and the activation of multiple interconnected oncogenic signaling pathways. These processes collectively favor epithelial plasticity, extracellular matrix remodeling, immune dysregulation, and establishment of a tumor-permissive pulmonary microenvironment. Nevertheless, definitive epidemiological evidence linking chronic inhalation exposure to lung cancer remains unavailable, and causality has not yet been established. Future investigations should therefore prioritize environmentally relevant long-term inhalation models, standardized exposure protocols, integrated multi-omics analyses, and well-designed prospective human studies to clarify the clinical significance of these mechanistic observations and to determine whether MNPs represent independent carcinogenic agents or environmental modifiers acting in concert with established pulmonary carcinogens [61,62,77].

## 6. Experimental Evidence from In Vitro and In Vivo Studies

Experimental models support the mechanistic framework described above across multiple biological scales.

In vitro systems consistently demonstrate size-, surface-, and dose-dependent internalization patterns of MNPs, accompanied by oxidative stress, mitochondrial dysfunction, DNA damage, inflammatory activation, and impaired barrier integrity [25,59,60]. Surface modifications may further modulate genotoxic potential [60].

Non-malignant lung epithelial cells frequently exhibit greater sensitivity than malignant lines [19,63,64], indicating that early-stage epithelium may represent a biologically vulnerable target. NPs also disrupt mitochondrial metabolism in bronchial smooth muscle cells [78], suggesting metabolic reprogramming as a cross-cellular phenomenon.

Mixture paradigms extend these findings. Co-exposure to phthalates [79] or cigarette smoke condensate [10] enhances ROS production, DNA damage, and transformation-associated phenotypes. Chronic low-dose exposure induces a gradual acquisition of carcinogenesis-related traits [80,81,82,83,84,85], aligning more closely with tumor promotion paradigms than acute toxicity paradigms.

Advanced airway constructs and transcriptomic platforms reveal coordinated dysregulation of oxidative stress networks, inflammatory pathways, and cell-cycle regulators [81,82]. In vivo exposure models demonstrate sterile inflammation, ER stress activation, ferroptosis [53], proteostasis disruption [83], microbiota-mediated metabolic alterations [84], and adhesion pathway destabilization (SUSD2–CLDN18.2 axis) linked to tumor progression [85] (Table 1).

Although heterogeneity in particle composition and exposure realism persists, the convergence of mechanistic endpoints across experimental platforms strengthens the overall argument for biological plausibility [19,58].

## 7. Human and Tissue Detection Studies

Human data on pulmonary exposure to MNPs remain limited but are gradually emerging. Occupational settings historically provide indirect evidence that the inhalation of synthetic polymer particles and fibers can be associated with respiratory symptoms and inflammatory lung conditions, particularly in industries involving textile production, plastic manufacturing, or high particulate exposure. Although these observations do not specifically isolate NPs, they support that the chronic inhalation of polymeric particles may have respiratory consequences.

Direct biomonitoring evidence has recently intensified this concern. A landmark investigation identified synthetic polymer particles—including polypropylene and polyethylene terephthalate—in surgically resected human lung tissue using spectroscopic techniques, providing early confirmation of microplastic deposition in the distal respiratory tract [86]. The localization of these particles within deep lung regions indicates that inhaled microplastics can bypass upper airway defenses and persist in alveolar compartments. Complementary post-mortem analyses further suggest MP bioaccumulation in human lungs and other organs, with smaller particles showing deeper parenchymal penetration and evidence of local tissue interaction [87]. These findings support pulmonary retention and raise concerns about sustained tissue exposure.

More recently, Zhao et al. reported the detection of MPs across multiple human tumor types, with lung cancer specimens demonstrating one of the highest detection frequencies [31,88]. Approximately 80% of analyzed lung tumor samples were positive for synthetic polymer particles, predominantly PP, PET, and PE-polymers widely used in textiles and consumer products and consistent with inhalational exposure pathways [88]. Importantly, particles were identified within the tumor tissue itself, indicating that inhaled microplastics may not only reach the lower respiratory tract, but also persist within malignant pulmonary microenvironments. However, the temporal relationship between particle accumulation and tumor development remains uncertain. Current human tissue studies are predominantly cross-sectional and therefore cannot determine whether MNPs accumulated before malignant transformation and potentially contributed to carcinogenesis, or whether pre-existing tumours—with altered tissue architecture, impaired clearance, increased vascular permeability, or local inflammatory changes—facilitated secondary particle retention. Both scenarios are biologically plausible, and they are not mutually exclusive. Consequently, the detection of MNPs within tumor tissue should be interpreted as evidence of particle accumulation rather than proof of a causal or chronological relationship with tumor development. The presence of smaller-sized particles further supports the plausibility of deep alveolar deposition and limited distal clearance [87,88]. Although the study does not establish a causal link between MP exposure and lung carcinogenesis, the high prevalence of particles in lung tumor tissues provides clinically relevant observational evidence of pulmonary accumulation at sites of malignant transformation.

Despite these advances, epidemiological evidence linking MNP exposure directly to lung cancer or other chronic pulmonary diseases remains sparse. Current human data primarily document presence and accumulation rather than clinical outcomes. No large-scale longitudinal studies have yet quantified dose–response relationships, exposure thresholds, or definitive cancer risk attributable to MNP inhalation. Thus, while biomonitoring studies indicate that inhaled plastic particles reach and persist in human lung tissue—and may even be detectable within tumor specimens—a substantial knowledge gap remains regarding their long-term pathological and oncogenic significance. Detection of polymeric material does not, in isolation, establish a causal association with disease development.

## 8. Comparison with Established Inhaled Carcinogens

The evaluation of inhaled MNPs gains interpretative strength when contextualized alongside well-established inhaled carcinogens such as asbestos, crystalline silica, diesel exhaust, and fine particulate matter (PM2.5). These agents are classified as Group 1 carcinogens by the International Agency for Research on Cancer (IARC) based on robust epidemiological and mechanistic evidence linking chronic inhalation to lung cancer [89,90,91,92].

A key shared feature is deep alveolar deposition and biopersistence. Despite their distinct chemical composition, MNPs and asbestos share several physicochemical characteristics that may contribute to similar pulmonary pathogenic mechanisms. Both materials may occur as elongated fibers or irregularly shaped particles capable of reaching the distal airways when their aerodynamic properties permit alveolar deposition. Small size, high aspect ratio in fibrous forms, resistance to complete pulmonary clearance, and prolonged tissue residence increase the likelihood of persistent interaction with alveolar epithelial cells and macrophages. In addition, both materials possess relatively large surface areas capable of interacting with biological molecules and adsorbing exogenous environmental contaminants, thereby influencing cellular responses. Nevertheless, important differences should be emphasized. Unlike asbestos, whose crystalline mineral structure, iron content, and intrinsic surface reactivity directly contribute to ROS generation and carcinogenicity, MNPs are chemically heterogeneous organic polymers that generally lack intrinsic redox catalytic activity. Consequently, although the two particle classes share several biophysical characteristics related to deposition, persistence, and chronic tissue interaction, their molecular mechanisms of toxicity are not identical and should not be regarded as equivalent. Asbestos fibers and respirable crystalline silica reach the distal lung and resist efficient clearance, leading to prolonged interaction with alveolar macrophages and epithelial cells [89,90]. This persistent retention is associated with sustained inflammatory signaling and oxidative stress—central drivers of particle-induced carcinogenesis. Similar deposition physics apply to ultrafine and nanoscale particles, which accumulate in the alveolar region due to diffusion-dominated transport [93]. Although polymeric NPs differ chemically from mineral fibers, their small size and potential for incomplete macrophage clearance raise comparable concerns regarding prolonged tissue residence.

Another mechanistic convergence involves chronic inflammation and redox imbalance. Asbestos and silica induce continuous macrophage activation, release of reactive oxygen and nitrogen species, and secretion of pro-inflammatory cytokines that contribute to DNA damage and aberrant tissue remodeling [89,90]. Diesel exhaust and PM2.5 similarly promote oxidative stress, epithelial injury, and the activation of proliferative signaling pathways associated with lung carcinogenesis [91,92]. Evidence from these established agents is that sustained, low-grade inflammation—rather than acute toxicity alone—constitutes an important contributor to malignant transformation.

Important distinctions must also be acknowledged. Unlike asbestos or crystalline silica, whose fibrous morphology and surface reactivity are directly linked to well-characterized pathogenic mechanisms (e.g., frustrated phagocytosis, fiber dimension-dependent carcinogenicity), MNPs exhibit highly heterogeneous shapes, polymer chemistries, and additive compositions. Moreover, definitive epidemiological dose–response data remain lacking for MNPs, whereas silica, asbestos, and diesel exhaust have decades of occupational and population-level evidence supporting causal inference [89,90,91,92]. Thus, while mechanistic parallels exist, equivalence in carcinogenic classification is not currently justified.

Lessons from established inhaled carcinogens provide a framework for future MNP research. In the case of asbestos and crystalline silica, lung cancer frequently manifests only after prolonged latency periods, often decades following initial exposure. Moreover, chronic exposure at relatively low concentrations can yield substantial cumulative biological impact. Particle–pollutant interactions further illustrate this complexity and influence toxicity, as observed in diesel exhaust and ambient particulate mixtures [91]. Collectively, these principles suggest that evaluating MNPs requires long-term exposure models, mixture toxicology approaches, and standardized exposure assessment strategies rather than reliance on short-term high-dose experimental designs.

In conclusion, MNPs cannot currently be categorized alongside established inhaled carcinogens in terms of confirmed carcinogenic potency. However, the shared mechanistic themes of deep lung deposition, persistent oxidative stress, inflammatory amplification, and epithelial remodeling provide biologically coherent grounds for continued investigation. Historical experience with asbestos, silica, and particulate air pollution underscores the importance of early mechanistic warning signals before definitive epidemiological proof emerges.

To facilitate comparison between inhaled MNPs and other well-established inhaled carcinogenic particulates, the principal biological mechanisms implicated in pulmonary carcinogenesis are summarized in Table 2.

## 9. Discussion

The rapid expansion of global plastic production has introduced a persistent class of inhalable particulates into the human environment, raising a central question: do MNPs primarily represent a detectable exposure, or a biologically meaningful modifier of lung carcinogenesis? Plastic pollution is now recognized as one of the defining environmental challenges of the 21st century [1], and the pulmonary system—by virtue of its vast surface area and direct environmental interface—constitutes a primary site of contact.

As detailed in Section 4, Section 5 and Section 6, inhaled MNPs converge on oxidative, inflammatory, and tumor-associated signaling pathways. These effects are frequently size-dependent and accentuated under chronic or co-exposure paradigms, suggesting that NPs may exert a disproportionate biological influence relative to larger MP fractions.

A useful contextual framework emerges when comparing MNPs with established inhaled carcinogens such as asbestos and crystalline silica. These materials do not induce cancer through direct genotoxicity alone; rather, their pathogenicity is mediated through biopersistence, chronic inflammation, frustrated phagocytosis, and sustained oxidative injury [4]. NPs share certain biokinetic properties—including efficient alveolar deposition and incomplete macrophage-mediated clearance [5]—that conceptually align with this mechanistic paradigm. However, unlike mineral fibers, polymeric particles lack inherent crystalline reactivity and rigid fibrogenic structure. This distinction suggests that if MNPs influence carcinogenesis, their role may be subtler and more modulatory than that of classical occupational carcinogens.

An increasingly plausible model is that MNPs function not as primary initiators, but as environmental co-factors. By sustaining low-grade oxidative stress, altering epithelial–immune crosstalk, and destabilizing barrier integrity, chronic NP exposure may reduce the threshold required for malignant transformation in the presence of other insults—such as tobacco smoke, air pollution, or pre-existing inflammatory lung disease. The interaction with co-contaminants adsorbed onto particle surfaces [2,3] further complicates attribution, as plastics may serve as delivery vehicles for mutagenic compounds, amplifying local toxicant burden within the alveolar niche.

**Figure 5:**
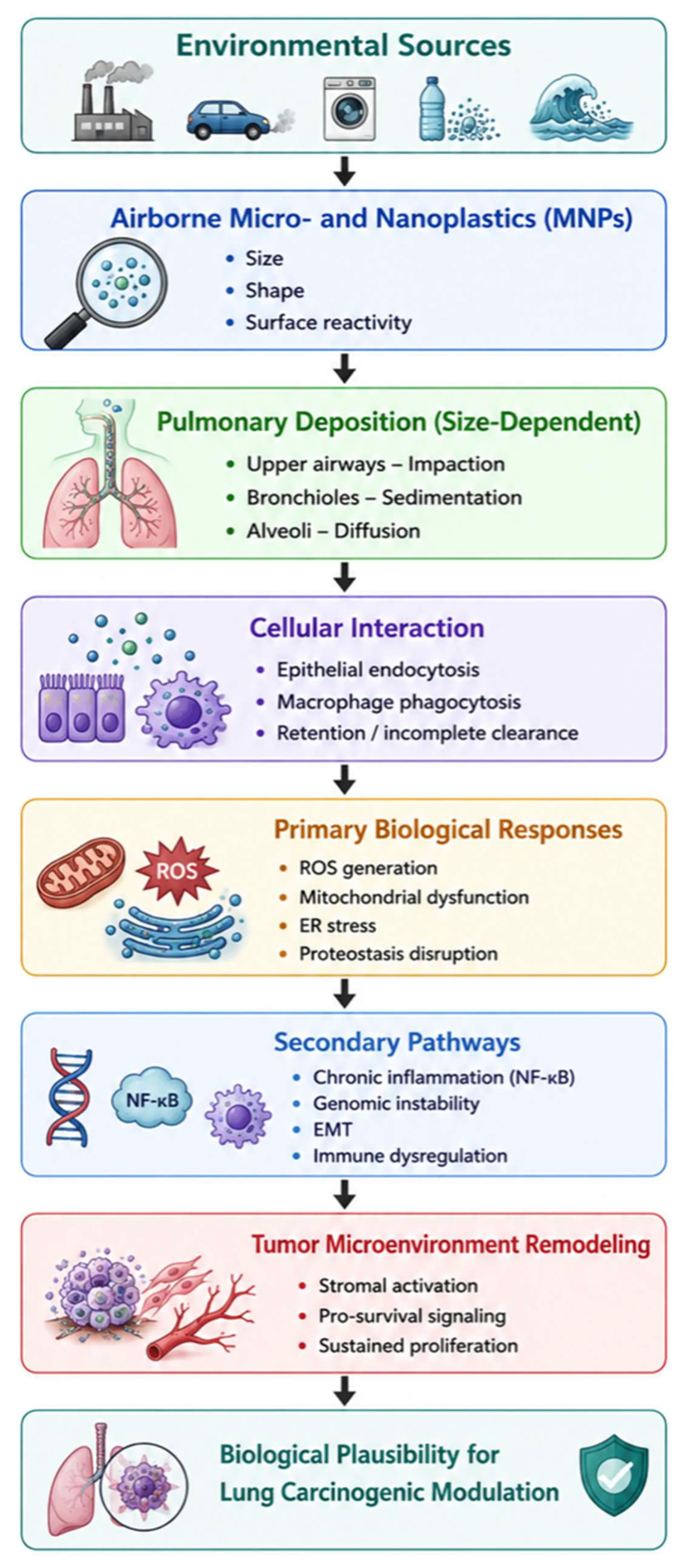
Integrated conceptual framework summarizing current experimental evidence linking inhaled MNPs to carcinogenesis-associated pathways. Size-dependent deposition in distal lung regions, incomplete clearance, and cellular internalization initiate oxidative stress and inflammatory signaling cascades. Persistent redox imbalance and immune modulation converge with established carcinogenic mechanisms, including genomic instability and tumor microenvironment remodeling. The model illustrates biological plausibility rather than confirmed causal classification.

An additional and increasingly relevant source of inhalational micro- and nanoplastic exposure is the use of electronic cigarettes. Recent studies have demonstrated that aerosols generated by e-cigarette devices may contain MNPs released from polymeric device components, including cartridges, mouthpieces, seals, and heating elements, through thermal degradation and mechanical wear. These particles are inhaled directly into the respiratory tract together with nicotine, flavoring compounds, metals, and other aerosol constituents, thereby representing a potentially overlooked source of pulmonary MNP exposure. Although the long-term clinical implications remain uncertain, the coexistence of MNPs with other inhaled toxicants may further amplify oxidative stress, chronic inflammation, and epithelial injury, reinforcing the concept that contemporary inhalational exposures frequently involve complex mixtures of interacting environmental contaminants rather than isolated agents [94,95] (Figure 5).

Biokinetically, the capacity of nanoparticles to traverse the air–blood barrier and distribute systemically [5] expands the conceptual impact beyond localized pulmonary injury. Persistent intracellular retention, incomplete clearance, and slow elimination kinetics increase the likelihood of cumulative tissue interaction under repeated exposure scenarios. Yet, whether such chronic redox perturbation is sufficient to drive clinically meaningful oncogenic progression in humans remains unresolved.

A major translational limitation is the disconnect between detection and disease correlation. The presence of plastic particles in human lung tissue confirms exposure but does not establish causality. Reliable exposure–response modeling will require standardized analytical methodologies, rigorous contamination control, and harmonized reporting frameworks, as emphasized in recent advances in biomonitoring and exposomics research [2]. In the absence of quantitative exposure metrics, longitudinal data, and validated biomarkers of effect, risk assessment remains speculative.

A broader implication is that MNPs may represent a new category of environmental modifier in carcinogenesis—diffuse, chronic, and globally ubiquitous. Unlike classical occupational carcinogens characterized by high-dose exposures in defined populations, plastic particulates are encountered at low levels across entire societies. Any potential impact may therefore be probabilistic rather than deterministic, influencing susceptibility, tumor progression, or treatment response rather than acting as singular causal agents. These findings should be interpreted within the constraints of experimental model systems and emerging exposure data.

### 9.1. Tumor Initiation Versus Tumor Promotion: Current Evidence

A critical question in assessing the carcinogenic relevance of inhaled MNPs is whether they function primarily as tumor initiators, directly inducing irreversible genetic damage, or as tumor promoters, reshaping tissue microenvironments to favor malignant progression.

Evidence for initiation remains suggestive but incomplete. In vitro studies demonstrate ROS generation, DNA strand breaks, chromosomal instability, and cell-cycle disruption following NP exposure [19,60]. However, most data derive from short-term experimental systems, often at supra-environmental concentrations, and direct demonstration of mutation fixation or tumor initiation in vivo remains lacking.

In contrast, the tumor promotion framework aligns more consistently with current findings. Inhaled MNPs exhibit characteristics typical of microenvironmental modifiers: persistent alveolar deposition, incomplete clearance, sustained oxidative stress, inflammasome activation, macrophage dysfunction, epithelial–mesenchymal transition (EMT), and synergy with co-exposures such as cigarette smoke and ozone [10,51,79]. Chronic low-dose exposure models further suggest a gradual acquisition of transformation-associated phenotypes rather than acute genotoxic collapse [82,85].

Taken together, the balance of mechanistic evidence more strongly supports a tumor-promoting and susceptibility-modifying role for inhaled MNPs than a confirmed primary initiating effect. This distinction is critical: rather than acting as classical high-potency carcinogens, MNPs may function as chronic inflammatory co-factors that lower the threshold for malignant transformation in already at-risk lung tissue.

Whether long-term exposure can bridge the gap between promotion and initiation remains an open question requiring mutation-specific, exposure-stratified, and longitudinal investigation.

### 9.2. Implications from a Thoracic Surgical Perspective

From a thoracic surgical perspective, inhaled MNPs introduce a potentially relevant environmental factor within the broader context of lung disease and carcinogenesis. Thoracic surgeons routinely manage patients whose pulmonary pathology reflects cumulative inhalational exposures, including tobacco smoke, particulate matter, and occupational dusts. The mechanistic parallels between these established exposures and MNPs—particularly alveolar deposition, biopersistence, and chronic inflammatory signaling—suggest that plastic particulates may represent an additional environmental modifier influencing surgical case profiles [4,89,90,91,92]. Importantly, the detection of microplastics in human lung tissue, including resected specimens [31,86], indicates that these particles are already present within the operative field. From a surgical oncology standpoint, this aligns with the evolving concept of the “exposome” as a determinant of lung cancer biology and patient heterogeneity [96,97,98].

Adequate preoperative assessment is central to thoracic surgical decision-making, particularly in determining operability and the extent of lung resection. Pulmonary function, cardiopulmonary reserve, and underlying parenchymal integrity are key determinants of postoperative outcomes. Chronic inflammatory and oxidative processes, such as those induced by MNP exposure [6,42,47], may contribute to subclinical impairment of lung function or exacerbate existing disease. Established evidence demonstrates that reduced pulmonary reserve and chronic lung injury are strongly associated with increased postoperative morbidity and mortality following lung resection [99,100]. Although direct clinical data on MNP exposure are lacking, their documented capacity to induce epithelial injury, macrophage dysfunction, and inflammatory remodeling suggests a plausible contribution to the cumulative burden of lung damage relevant to surgical risk stratification. This may be particularly relevant in borderline operability scenarios where small differences in lung function can influence the choice between lobectomy, sublobar resection, atypical extra-anatomical resections, or non-surgical treatment.

Intraoperatively, thoracic surgeons frequently encounter variability in tissue characteristics that reflect underlying environmental and inflammatory exposures. Chronic inhalational insults are known to result in pulmonary fibrosis, pleural thickening, and altered tissue planes, which may increase technical complexity and operative time. Mechanistic studies demonstrating macrophage activation, oxidative stress, and persistent inflammatory signaling in response to MNPs [48,49] are consistent with pathways implicated in fibrotic remodeling. Analogous effects have long been recognized in conditions such as pneumoconiosis and asbestos-related diseases, where tissue rigidity and adhesions complicate resection [89,90]. In addition, tumor microenvironment alterations, such as inflammation-driven stromal remodeling and immune modulation, have been shown to influence tumor consistency and surgical handling [101,102]. The reported presence of MPs within tumor tissue [31,88] raises the possibility that such particles may interact with the tumor microenvironment, although the clinical implications for surgical technique remain to be determined.

Thoracic surgeons also play a critical role in advancing translational and tissue-based research. Resected lung specimens provide a unique opportunity for the direct assessment of environmental exposures within human tissue. Previous studies have demonstrated the feasibility of detecting MPs in surgically obtained lung samples using spectroscopic techniques [31,86]. Incorporation of standardized sampling and analytical protocols into surgical workflows could enable the correlation of particle burden with histopathological features, inflammatory markers, and clinical outcomes. This approach parallels established translational research models in thoracic oncology, where resected specimens have been instrumental in advancing molecular profiling and biomarker discovery [103]. Given the current lack of epidemiological data, such tissue-based investigations may represent a critical step toward bridging the gap between exposure detection and clinical relevance.

Finally, the broader role of the thoracic surgeon within multidisciplinary cancer care and multimodality treatment pathways underlines the importance of integrating emerging environmental risk factors into clinical awareness. Lung cancer progression, recurrence, and treatment response are increasingly understood to be influenced by the tumor microenvironment and systemic inflammatory state. Experimental evidence linking MNP exposure to epithelial–mesenchymal transition, immune dysregulation, and tumor-promoting signaling pathways [7,50,57] suggests potential relevance beyond tumor initiation alone. Chronic inflammation and immune modulation have been shown to affect both surgical outcomes and long-term oncologic prognosis [104,105,106]. While current evidence does not support direct clinical decision-making based on MNP exposure, awareness of such environmental modifiers may become increasingly relevant in-patient counseling, risk stratification, and future precision medicine approaches in thoracic oncology.

## 10. Limitations

Several limitations must be acknowledged when interpreting the conclusions of this review. Direct epidemiological evidence linking inhaled MNPs to lung cancer in humans remains absent. Current human data are limited primarily to biomonitoring and tissue-detection studies, which confirm exposure and deposition but do not establish exposure–response relationships or cancer incidence risk.

Substantial heterogeneity exists across experimental studies. Variations in particle size, polymer type, surface modification, aging/weathering status, shape (spherical vs. fibrous), and exposure duration limit direct comparison. Many studies rely on pristine, commercially manufactured polystyrene spheres, which may not accurately represent environmentally aged, chemically complex MNPs encountered in real-world settings.

Exposure metrics also vary widely, with doses expressed as mass concentration, particle number, or surface area. This lack of standardized dose metrics complicates extrapolation to human inhalation scenarios and impedes quantitative risk modeling. In addition, definitions of nanoplastics remain non-uniform, with size thresholds varying between <100 nm and <1000 nm across publications. This variability affects classification, toxicological interpretation, and cross-study comparability.

Environmentally relevant chronic low-dose exposure models remain limited. Many in vitro experiments employ concentrations exceeding the estimated ambient exposure levels, raising concerns regarding dose realism and translational relevance. Long-term cumulative exposure paradigms are still underdeveloped.

Mixture effects further complicate interpretation. MNPs coexist with tobacco smoke, diesel exhaust, ozone, particulate matter, and adsorbed environmental toxicants. Disentangling independent versus synergistic effects remains methodologically complex.

Analytical limitations also warrant attention, including potential sample contamination, incomplete sensitivity for nanoscale detection, and the lack of harmonized reporting frameworks, which further complicates contamination control and data comparability. These constraints may contribute to the underestimation or misclassification of exposure.

Finally, as a narrative review, selection bias and publication bias cannot be excluded, despite efforts to include mechanistically diverse and balanced evidence. In addition, many experimental investigations rely on laboratory-generated particles under controlled conditions, which may not fully replicate environmentally aged and compositionally heterogeneous materials. Collectively, these limitations underscore that current conclusions support biological plausibility rather than confirmed carcinogenic classification.

## 11. Quantitative Exposure and Dose Uncertainty

Although mechanistic evidence for pulmonary toxicity is expanding, quantitative characterization of inhalational exposure to MNPs remains limited. Ambient concentrations vary widely depending on geographic region sampling methodology and environmental context. Estimates of airborne microplastic levels are often reported in particles per cubic meter; however, translation into biologically relevant lung dose remains challenging due to variability in aerodynamic diameter, deposition fraction, and clearance kinetics. Reported environmental concentrations vary substantially depending on sampling methodology, geographic location, and particle size cutoffs.

Many experimental studies employ exposure concentrations that may exceed the currently reported environmental levels, raising important questions regarding dose realism and risk extrapolation. Furthermore, standardized metrics for nanoplastic quantification—such as particle number, surface area, mass concentration, or polymer-specific burden—are not harmonized across studies. This heterogeneity complicates cross-comparison and limits accurate exposure–response modeling.

Bridging this gap requires the integration of aerosol physics modeling, realistic chronic inhalation systems, and quantitative particle burden measurements in lung tissue. Without harmonized dose metrics and longitudinal exposure data, definitive risk characterization for lung cancer remains premature despite strong biological plausibility.

## 12. Future Perspectives/Research Directions

The central challenge for the coming decade is not to prematurely classify MNPs as lung carcinogens, but to rigorously determine where they lie along the spectrum between benign environmental residue and biologically active risk modifier. The convergence of oxidative stress induction [6,96], inflammatory amplification [4,96], incomplete alveolar clearance [5], and alignment with canonical cancer pathways [7,96] supports mechanistic plausibility. However, plausibility alone is insufficient for public health classification.

Future research must integrate realistic chronic exposure models, quantitative particle burden assessment, multi-omics profiling, and long-term epidemiological surveillance. Only through such integrative approaches can we discern whether inhaled MNPs subtly reshape the pulmonary microenvironment in a manner that meaningfully contributes to lung carcinogenesis—or whether their biological footprint remains below pathological relevance.

In the context of an increasingly plastic-saturated world, this distinction is not merely academic. It will determine whether MNPs are viewed as a transient toxicological concern or as a defining environmental modifier of respiratory disease in the Anthropocene.

## 13. Conclusions

Inhaled micro- and nanoplastics have emerged as ubiquitous environmental contaminants with the potential to influence multiple biological processes implicated in lung carcinogenesis. Current experimental evidence consistently demonstrates that MNP exposure induces oxidative stress, chronic inflammation, mitochondrial dysfunction, genomic instability, epigenetic alterations, and the dysregulation of several cancer-related signaling pathways, thereby providing a biologically plausible framework for their involvement in tumor promotion. Nevertheless, definitive evidence supporting a causal relationship between chronic MNP inhalation and human lung cancer remains lacking, and current knowledge is derived predominantly from experimental and mechanistic studies. Future research should prioritize standardized exposure assessment, environmentally relevant long-term inhalation models, integrated multi-omics approaches, and well-designed epidemiological investigations to determine the true clinical significance of MNP exposure. Until such evidence becomes available, MNPs should be regarded as promising but unconfirmed environmental modifiers of lung carcinogenesis rather than established human pulmonary carcinogens.

## Figures and Tables

**Table 1:** Summary of representative experimental, co-exposure, and biomonitoring studies evaluating pulmonary responses to inhaled MNPs. Evidence spans in vitro mechanistic investigations, in vivo exposure models, and human tissue detection studies. While causality for lung cancer remains unproven, mechanistic convergence with recognized carcinogenic pathways—including oxidative stress, chronic inflammation, proliferative signaling, and tumor microenvironment modulation—is evident.

Study [Ref]	Model	Particle Type/Size	Exposure Design	Key Findings	Carcinogenic Relevance
Xu et al. [59]	A549 lung epithelial cells	Polystyrene nanoplastics	Acute in vitro exposure	Cellular internalization, ROS production	Initiation of oxidative DNA damage
Shi et al. [60]	A549 cells	PS micro- and nanoplastics (size-dependent)	In vitro dose–response	Cytotoxicity and genotoxicity	Genomic instability
Ernhofer et al. [19]	Non-malignant lung epithelial cells	PS nanoparticles	In vitro exposure	DNA damage, oxidative stress, mitogenic signaling	Proliferative signaling activation
Milillo et al. [52]	Human alveolar epithelial cell line	PS nanoplastics	In vitro exposure	Oxidative stress, senescence, apoptosis	Chronic epithelial injury
Wu et al. [53]	Murine lung model	PS nanoplastics	In vivo exposure	ROS-dependent ER stress, apoptosis, ferroptosis	Persistent redox imbalance
Han et al. [35]	Lung epithelial cells	PS nanoplastics	In vitro mechanistic study	Integrin α5β1-mediated endocytosis	Receptor-mediated uptake pathway
Laganà et al. [30]	Pulmonary cell models	Micro-/nanopolystyrene	In vitro exposure	Sterile inflammation	Chronic inflammatory microenvironment
Jian et al. [51]	Airway inflammation model	PS nanoplastics + ozone	Co-exposure	Synergistic inflammatory amplification	Tumor promotion context
Morataya-Reyes et al. [10]	In vitro PET + cigarette smoke	PET nanoplastics	Long-term co-exposure	Enhanced carcinogenic traits	Co-exposure synergy
Wang et al. [85]	Chronic low-dose exposure model	Nanoplastics	Long-term exposure	SUSD2–CLDN18.2 dysregulation	Lung adenocarcinoma progression
Amato-Lourenço et al. [86]	Human lung tissue	Airborne microplastics	Biomonitoring	Microplastic detection in lung parenchyma	Exposure confirmation
Zhao et al. [31]	Human tumor tissues	Microplastics	Tissue analysis	Microplastics in tumor samples	Potential accumulation relevance

**Table 2:** Comparison of the biological characteristics and carcinogenesis-related mechanisms of inhaled micro- and nanoplastics (MNPs) and established inhaled carcinogenic particulates. The table summarizes current evidence regarding the major biological processes implicated in pulmonary carcinogenesis following inhalation exposure. Unlike asbestos, crystalline silica, and diesel exhaust particles, MNPs have not been classified as human carcinogens, and current evidence derives predominantly from experimental studies. Nevertheless, substantial mechanistic overlap exists with established inhaled carcinogenic particulates, particularly with respect to oxidative stress, chronic inflammation, genomic instability, mitochondrial dysfunction, epithelial–mesenchymal transition, and persistent tissue remodeling. The ability of MNPs to adsorb and transport environmental contaminants represents an additional characteristic that may further modulate their biological activity.

Feature	Micro-/Nanoplastics (MNPs)	Asbestos	Crystalline Silica	Diesel Exhaust Particles (DEP)
Primary exposure route	Environmental and occupational inhalation	Occupational inhalation	Occupational inhalation	Environmental and occupational inhalation
Particle persistence	High (polymer-dependent)	Very high	Very high	Moderate
Deep alveolar deposition	Yes (especially nanoplastics)	Yes	Yes	Yes
Macrophage dysfunction	Yes	Yes	Yes	Yes
Oxidative stress	Strong experimental evidence	Well-established	Well-established	Well-established
Chronic inflammation	Strong experimental evidence	Established	Established	Established
DNA damage/genomic instability	Experimental evidence	Established	Established	Established
Epigenetic alterations	Emerging evidence	Reported	Reported	Reported
Mitochondrial dysfunction	Experimental evidence	Reported	Reported	Reported
EMT induction	Experimental evidence	Reported	Reported	Reported
Fibrosis	Observed experimentally	Major feature	Major feature	Limited
Carrier for environmental toxicants	Major characteristic	Limited	Minimal	Adsorbs organic compounds
Direct human epidemiological evidence for lung cancer	Not yet established	Strong	Strong	Strong
IARC carcinogenic classification	Not classified	Group 1	Group 1	Group 1
Overall evidence for carcinogenicity	Mechanistically plausible; experimental evidence predominates	Established human carcinogen	Established human carcinogen	Established human carcinogen

## Data Availability

No new data were created or analyzed in this study.

## Abbreviations

MNPs	Micro- and nanoplastics
MPs	Microplastics
NPs	Nanoplastics
ROS	Reactive oxygen species
EMT	Epithelial–mesenchymal transition
ER	Endoplasmic reticulum
PM_2_._5_	Particulate matter with aerodynamic diameter ≤2.5 μm
PAHs	Polycyclic aromatic hydrocarbons
PCBs	Polychlorinated biphenyls
PBDEs	Polybrominated diphenyl ethers
PFAS	Per- and polyfluoroalkyl substances
BPA	Bisphenol A
PFCAs	Perfluorocarboxylic acids
FTIR	Fourier transform infrared spectroscopy
TEM	Transmission electron microscopy
SEM	Scanning electron microscopy
AFM	Atomic force microscopy
LDIR	Laser direct infrared imaging
O-PTIR	Optical photothermal infrared spectroscopy
Py-GC-MS	Pyrolysis-gas chromatography-mass spectrometry
TGA	Thermogravimetric analysis
IARC	International Agency for Research on Cancer
MAPK	Mitogen-activated protein kinase
PI3K/Akt	Phosphoinositide 3-kinase/Protein kinase B
ATII	Alveolar type II cells
NLRP3	NOD-like receptor family pyrin domain containing 3
IL-1β	Interleukin-1 beta
TNF-α	Tumor necrosis factor alpha
NF-κB	Nuclear factor kappa B

## References

[1] Landrigan P.J., Raps H., Cropper M., Bald C., Brunner M., Canonizado E.M., Charles D., Chiles T.C., Donohue M.J., Enck J. (2023). The Minderoo-Monaco Commission on Plastics and Human Health. Ann. Glob. Health.

[2] Vasse G.F., Melgert B.N. (2024). Microplastic and plastic pollution: Impact on respiratory disease and health. Eur. Respir. Rev..

[3] Evangeliou N., Grythe H., Klimont Z., Heyes C., Eckhardt S., Lopez-Aparicio S., Stohl A. (2020). Atmospheric transport is a major pathway of microplastics to remote regions. Nat. Commun..

[4] Oberdörster G., Maynard A., Donaldson K., Castranova V., Fitzpatrick J., Ausman K., Carter J., Karn B., Kreyling W., Lai D. (2005). Principles for characterizing the potential human health effects from exposure to nanomaterials: Elements of a screening strategy. Part. Fibre Toxicol..

[5] Lu K., Zhan D., Fang Y., Li L., Chen G., Chen S., Wang L. (2022). Microplastics, potential threat to patients with lung diseases. Front. Toxicol..

[6] Leivaditis V., Sarafis P., Malliarou M., Shaska E., Dogjani A., Mulita F., Sergentanis T.N. (2025). Comprehensive analysis of natural and man-made disasters: A narrative review of causes, impacts and management strategies in greece and the european union. Surg. Chron..

[7] Edirisinghe D.T., Kaur J., Lee Y.Q., Lim H.X., Lo S.W.T., Vishupriyaa S., Tan E.W., Wong R.S.Y., Goh B.H. (2025). The role of the tumour microenvironment in lung cancer and its therapeutic implications. Med. Oncol..

[8] Thompson R.C., Olsen Y., Mitchell R.P., Davis A., Rowland S.J., John A.W., McGonigle D., Russell A.E. (2004). Lost at sea: Where is all the plastic?. Science.

[9] Ali N., Katsouli J., Marczylo E.L., Gant T.W., Wright S., Bernardino de la Serna J. (2024). The potential impacts of micro-and-nano plastics on various organ systems in humans. EBioMedicine.

[10] Morataya-Reyes M., Villacorta A., Gutiérrez-García J., Egea R., Martín-Pérez J., Barguilla I., Marcos R., Hernández A. (2025). The long-term in vitro co-exposure of polyethylene terephthalate (PET) nanoplastics and cigarette smoke condensate exacerbates the induction of carcinogenic traits. J. Hazard. Mater..

[11] Salvia R., Cañaveras M., Rico L.G., Drozdowskyj A., Ward M.D., Jurado R., Gómez-Muñoz L., Vives-Pi M., Martínez-Cáceres E., Petriz J. (2024). Prospective investigation of nanoplastic accumulation in healthy subjects, autoimmune diseases, hematological malignancies, lung cancer, and murine models. Microplastics.

[12] Deng X., Gui Y., Zhao L. (2025). The micro(nano)plastics perspective: Exploring cancer development and therapy. Mol. Cancer.

[13] Yee M.S., Hii L.W., Looi C.K., Lim W.M., Wong S.F., Kok Y.Y., Tan B.K., Wong C.Y., Leong C.O. (2021). Impact of Microplastics and Nanoplastics on Human Health. Nanomaterials.

[14] Camia B., Casasco A., Monti M. (2025). Forever particles: Histochemistry in the plasticene age. Eur. J. Histochem..

[15] Delaney S., Rodriguez C., Sarrett S.M., Dayts E.J., Zeglis B.M., Keinänen O. (2023). Unraveling the in vivo fate of inhaled micro- and nanoplastics with PET imaging. Sci. Total Environ..

[16] Ruggieri L., Amato O., Marrazzo C., Nebuloni M., Dalu D., Cona M.S., Gambaro A., Rulli E., La Verde N. (2024). Rising Concern About the Carcinogenetic Role of Micro-Nanoplastics. Int. J. Mol. Sci..

[17] Mohamed Nor N.H., Koelmans A.A. (2019). Transfer of PCBs from Microplastics under Simulated Gut Fluid Conditions Is Biphasic and Reversible. Environ. Sci. Technol..

[18] Jenner L.C., Rotchell J.M., Bennett R.T., Cowen M., Tentzeris V., Sadofsky L.R. (2022). Detection of microplastics in human lung tissue using μFTIR spectroscopy. Sci. Total Environ..

[19] Ernhofer B., Spittler A., Ferk F., Mišík M., Zylka M.M., Glatt L., Boettiger K., Solta A., Kirchhofer D., Timelthaler G. (2025). Small Particles, Big Problems: Polystyrene nanoparticles induce DNA damage, oxidative stress, migration, and mitogenic pathways predominantly in non-malignant lung cells. J. Hazard. Mater..

[20] Vincoff S., Schleupner B., Santos J., Morrison M., Zhang N., Dunphy-Daly M.M., Eward W.C., Armstrong A.J., Diana Z., Somarelli J.A. (2024). The Known and Unknown: Investigating the Carcinogenic Potential of Plastic Additives. Environ. Sci. Technol..

[21] Dzierżyński E., Gawlik P.J., Puźniak D., Flieger W., Jóźwik K., Teresiński G., Forma A., Wdowiak P., Baj J., Flieger J. (2024). Microplastics in the Human Body: Exposure, Detection, and Risk of Carcinogenesis: A State-of-the-Art Review. Cancers.

[22] Mitrano D.M., Wick P., Nowack B. (2021). Placing nanoplastics in the context of global plastic pollution. Nat. Nanotechnol..

[23] Sychowski G., Romanowicz H., Cieślik-Wolski B., Wojciechowska-Durczyńska K., Smolarz B. (2025). Microplastics Exposure Impact on Lung Cancer—Literature Review. Cancers.

[24] Shao K., Zou R., Zhang Z., Mandemaker L.D.B., Timbie S., Smith R.D., Durkin A.M., Dusza H.M., Meirer F., Weckhuysen B.M. (2025). Advancements in Assays for Micro- and Nanoplastic Detection: Paving the Way for Biomonitoring and Exposomics Studies. Annu. Rev. Pharmacol. Toxicol..

[25] Zhang H., Zhang S., Duan Z., Wang L. (2022). Pulmonary toxicology assessment of polyethylene terephthalate nanoplastic particles in vitro. Environ. Int..

[26] Zha H., Xia J., Wang K., Xu L., Chang K., Li L. (2024). Foodborne and airborne polyethersulfone nanoplastics respectively induce liver and lung injury in mice: Comparison with microplastics. Environ. Int..

[27] Eberhard T., Casillas G., Zarus G.M., Barr D.B. (2024). Systematic review of microplastics and nanoplastics in indoor and outdoor air: Identifying a framework and data needs for quantifying human inhalation exposures. J. Expo. Sci. Environ. Epidemiol..

[28] Stapleton P.A. (2019). Toxicological considerations of nano-sized plastics. AIMS Environ. Sci..

[29] Lehner R., Weder C., Petri-Fink A., Rothen-Rutishauser B. (2019). Emergence of Nanoplastic in the Environment and Possible Impact on Human Health. Environ. Sci. Technol..

[30] Antonio L., Visalli G., Facciolà A., Saija C., Bertuccio M.P., Baluce B., Celesti C., Iannazzo D., Di Pietro A. (2024). Sterile inflammation induced by respirable micro and nano polystyrene particles in the pathogenesis of pulmonary diseases. Toxicol. Res..

[31] Zhao J., Zhang H., Shi L., Jia Y., Sheng H. (2024). Detection and quantification of microplastics in various types of human tumor tissues. Ecotoxicol. Environ. Saf..

[32] Winkler A.S., Cherubini A., Rusconi F., Santo N., Madaschi L., Pistoni C., Moschetti G., Sarnicola M.L., Crosti M., Rosso L. (2022). Human airway organoids and microplastic fibers: A new exposure model for emerging contaminants. Environ. Int..

[33] Feng Y., Tu C., Li R., Wu D., Yang J., Xia Y., Peijnenburg W.J.G.M., Luo Y. (2023). A systematic review of the impacts of exposure to micro- and nano-plastics on human tissue accumulation and health. Eco-Environ. Health.

[34] Michelini S., Mawas S., Kurešepi E., Barbero F., Šimunović K., Miremont D., Devineau S., Schicht M., Ganin V., Haugen Ø.P. (2025). Pulmonary hazards of nanoplastic particles: A study using polystyrene in in vitro models of the alveolar and bronchial epithelium. J. Nanobiotechnology.

[35] Han M., Liang J., Wang K., Si Q., Zhu C., Zhao Y., Khan N.A.K., Abdullah A.L.B., Shau-Hwai A.T., Li Y.M. (2024). Integrin A5B1-mediated endocytosis of polystyrene nanoplastics: Implications for human lung disease and therapeutic targets. Sci. Total Environ..

[36] Saha S.C., Saha G. (2024). Effect of microplastics deposition on human lung airways: A review with computational benefits and challenges. Heliyon.

[37] Rudolph J., Völkl M., Jérôme V., Scheibel T., Freitag R. (2021). Noxic effects of polystyrene microparticles on murine macrophages and epithelial cells. Sci. Rep..

[38] Giordano M.E., Lionetto F., Lionetto M.G. (2025). Impact of polyethylene terephthalate nanoplastics (PET) on fibroblasts: A study on NIH-3T3 cells. Front. Physiol..

[39] Green T.R., Fisher J., Stone M., Wroblewski B.M., Ingham E. (1998). Polyethylene particles of a ‘critical size’ are necessary for the induction of cytokines by macrophages in vitro. Biomaterials.

[40] Geiser M., Kreyling W.G. (2010). Deposition and biokinetics of inhaled nanoparticles. Part. Fibre Toxicol..

[41] Kaluç N., Bertorello S., Tombul O.K., Baldi S., Nannini G., Bartolucci G., Niccolai E., Amedei A. (2024). Gut-lung microbiota dynamics in mice exposed to Nanoplastics. NanoImpact.

[42] Cheng Y., Yang Y., Bai L., Cui J. (2024). Microplastics: An often-overlooked issue in the transition from chronic inflammation to cancer. J. Transl. Med..

[43] Reuter S., Gupta S.C., Chaturvedi M.M., Aggarwal B.B. (2010). Oxidative stress, inflammation, and cancer: How are they linked?. Free Radic. Biol. Med..

[44] Zeng S., Zhang Y., Li S., Li Z., Li P., Xie J., Zhang J., Xie L., Yang Y. (2025). From metabolic alterations to chronic inflammation: Mechanisms and immunoregulation of metabolic reprogramming in COPD. Front. Immunol..

[45] Cao Y., Zhao Q., Jiang F., Geng Y., Song H., Zhang L., Li C., Li J., Li Y., Hu X. (2024). Interactions between inhalable aged microplastics and lung surfactant: Potential pulmonary health risks. Environ. Res..

[46] Shi W., Cao Y., Chai X., Zhao Q., Geng Y., Liu D., Tian S. (2022). Potential health risks of the interaction of microplastics and lung surfactant. J. Hazard. Mater..

[47] Huang Y., Shang P., Li Y., Wang Y. (2025). Lung hazards of microplastics and their toxicological mechanisms. Environ. Pollut..

[48] Bazina L., Deloid G.M., Calderon L., Fritzky L., Vaze N., Tsiodra I., Mihalopoulos N., Pyrsopoulos T., Demokritou P. (2026). Impact of nanoplastics emitted from incineration of polyethylene plastic on THP-1 macrophage viability and immune function. J. Hazard. Mater..

[49] Florance I., Chandrasekaran N., Gopinath P.M., Mukherjee A. (2022). Exposure to polystyrene nanoplastics impairs lipid metabolism in human and murine macrophages in vitro. Ecotoxicol. Environ. Saf..

[50] Choudhury A., Simnani F.Z., Singh D., Patel P., Sinha A., Nandi A., Ghosh A., Saha U., Kumari K., Jaganathan S.K. (2023). Atmospheric microplastic and nanoplastic: The toxicological paradigm on the cellular system. Ecotoxicol. Environ. Saf..

[51] Jian X., Zhang X., Chang S., Xue Y., Shang P., Liu Y., Chen H., Zhou X., Wang W., Wang P. (2025). Co-exposure of polystyrene nanoplastics and ozone synergistically induced airway inflammation: Evidence and biomarkers screening. Ecotoxicol. Environ. Saf..

[52] Milillo C., Aruffo E., Di Carlo P., Patruno A., Gatta M., Bruno A., Dovizio M., Marinelli L., Dimmito M.P., Di Giacomo V. (2024). Polystyrene nanoplastics mediate oxidative stress, senescence, and apoptosis in a human alveolar epithelial cell line. Front. Public Health.

[53] Wu Q., Liu C., Liu D., Wang Y., Qi H., Liu X., Zhang Y., Chen H., Zeng Y., Li J. (2024). Polystyrene nanoplastics-induced lung apoptosis and ferroptosis via ROS-dependent endoplasmic reticulum stress. Sci. Total Environ..

[54] Gosselink I.F., van Schooten F.J., Drittij M.J., Höppener E.M., Leonhardt P., Moschini E., Serchi T., Gutleb A.C., Kooter I.M., Remels A.H. (2024). Assessing toxicity of amorphous nanoplastics in airway- and lung epithelial cells using air-liquid interface models. Chemosphere.

[55] Le T.K., Kuo C.H., Wu C.C., Chen Y.T., Hsieh S.L. (2026). Polystyrene nanoplastics and lung cancer: Insights from network toxicology and mechanistic in vitro studies. Toxicol. Appl. Pharmacol..

[56] Siemiątkowska B., Szczepanowska J. (2026). Mitochondrial stress response in lung cells triggered by the inhaled nanoplastics. Arch. Toxicol..

[57] Mishra S.K., Sanyal T., Kundu P., Kumar R., Ghosh D., Chakrabarti G., Sikdar N., Bhattacharya S., Paul S., Das A. (2025). Microplastics as emerging carcinogens: From environmental pollutants to oncogenic drivers. Mol. Cancer.

[58] Dong C.D., Chen C.W., Chen Y.C., Chen H.H., Lee J.S., Lin C.H. (2020). Polystyrene microplastic particles: In vitro pulmonary toxicity assessment. J. Hazard. Mater..

[59] Xu M., Halimu G., Zhang Q., Song Y., Fu X., Li Y., Li Y., Zhang H. (2019). Internalization and toxicity: A preliminary study of effects of nanoplastic particles on human lung epithelial cell. Sci. Total Environ..

[60] Shi X., Wang X., Huang R., Tang C., Hu C., Ning P., Wang F. (2022). Cytotoxicity and Genotoxicity of Polystyrene Micro- and Nanoplastics with Different Size and Surface Modification in A549 Cells. Int. J. Nanomed..

[61] Luo D., Chu X., Wu Y., Wang Z., Liao Z., Ji X., Ju J., Yang B., Chen Z., Dahlgren R. (2024). Micro- and nano-plastics in the atmosphere: A review of occurrence, properties and human health risks. J. Hazard. Mater..

[62] He Z., Shen Z., Zhang H., Na C., Bai C. (2025). From exposure to oncogenesis: A review on the multifaceted roles of microplastics in tumor initiation and progression. J. Transl. Med..

[63] Kim Y., Park S.J., Oh J.M. (2026). Polystyrene nanoplastics increase migration in normal lung cells while inducing differential cytotoxicity in lung cancer cells. Toxicology.

[64] Jahedi F., Jaafarzadeh Haghighi Fard N. (2025). Micro- and nanoplastic toxicity in humans: Exposure pathways, cellular effects, and mitigation strategies. Toxicol. Rep..

[65] Møller P., Roursgaard M. (2023). Exposure to nanoplastic particles and DNA damage in mammalian cells. Mutat. Res. Rev. Mutat. Res..

[66] Płuciennik K., Sicińska P., Misztal W., Bukowska B. (2024). Important Factors Affecting Induction of Cell Death, Oxidative Stress and DNA Damage by Nano- and Microplastic Particles In Vitro. Cells.

[67] Khan A., Jia Z. (2023). Recent insights into uptake, toxicity, and molecular targets of microplastics and nanoplastics relevant to human health impacts. iScience.

[68] Yang Z., DeLoid G.M., Zarbl H., Baw J., Demokritou P. (2023). Micro- and nanoplastics (MNPs) and their potential toxicological outcomes: State of science, knowledge gaps and research needs. NanoImpact.

[69] Domenech J., Annangi B., Marcos R., Hernández A., Catalán J. (2023). Insights into the potential carcinogenicity of micro- and nano-plastics. Mutat. Res. Rev. Mutat. Res..

[70] Ji Y., Chen L., Wang Y., Zhang J., Yu Y., Wang M., Wang X., Liu W., Yan B., Xiao L. (2024). Realistic Nanoplastics Induced Pulmonary Damage via the Crosstalk of Ferritinophagy and Mitochondrial Dysfunction. ACS Nano.

[71] Zhang S., Zhang H., Li Y., Sun Z., Chen Y. (2024). Recent advances on transport and transformation mechanism of nanoplastics in lung cells. Sci. Total Environ..

[72] Casella C., Ballaz S.J. (2024). Genotoxic and neurotoxic potential of intracellular nanoplastics: A review. J. Appl. Toxicol..

[73] Chen Y., Zhang Z., Ji K., Zhang Q., Qian L., Yang C. (2025). Role of microplastics in the tumor microenvironment (Review). Oncol. Lett..

[74] Wen J., Lin Y. (2025). Invisible invaders: Unveiling the carcinogenic threat of microplastics and nanoplastics in colorectal cancer-a systematic review. Front. Public Health.

[75] Nivedita J., Megha P., Daisy P.S., Sivachandran R., Ramprasath A., Rajkumar M., Anitha T.S. (2025). Microplastics and Cancer: A Comprehensive Review of Their Impact on Tumor Progression and Mechanisms of Carcinogenesis. J. Environ. Pathol. Toxicol. Oncol..

[76] Alaraby M., Abass D., Marcos R., Hernández A. (2026). Emerging evidence on micro- and nanoplastics carcinogenicity: Mechanisms, models, and signaling networks. Mol. Cancer.

[77] Kurhaluk N., Kołodziejska R., Rymuszka A., Bilski R., Kaczorowska-Bilska K., Tomin V., Kamiński P., Tkaczenko H. (2026). Microplastics as Vectors Influencing Oxidative Stress, Inflammation, and Endocrine Function During Early Development. Int. J. Mol. Sci..

[78] Winiarska E., Chaszczewska-Markowska M., Ghete D., Jutel M., Zemelka-Wiacek M. (2024). Nanoplastics Penetrate Human Bronchial Smooth Muscle and Small Airway Epithelial Cells and Affect Mitochondrial Metabolism. Int. J. Mol. Sci..

[79] Shi Q., Tang J., Wang L., Liu R., Giesy J.P. (2021). Combined cytotoxicity of polystyrene nanoplastics and phthalate esters on human lung epithelial A549 cells and its mechanism. Ecotoxicol. Environ. Saf..

[80] Gutiérrez-García J., Egea R., Barguilla I., Nymark P., García-Rodríguez A., Guyot B., Maguer-Satta V., Marcos R., Rubio L., Hernández A. (2025). Long-Term Exposure to Real-Life Polyethylene Terephthalate Nanoplastics Induces Carcinogenesis In Vitro. Environ. Sci. Technol..

[81] Busch M., Brouwer H., Aalderink G., Bredeck G., Kämpfer A.A.M., Schins R.P.F., Bouwmeester H. (2023). Investigating nanoplastics toxicity using advanced stem cell-based intestinal and lung in vitro models. Front. Toxicol..

[82] Yang S., Cheng Y., Chen Z., Liu T., Yin L., Pu Y., Liang G. (2021). In vitro evaluation of nanoplastics using human lung epithelial cells, microarray analysis and co-culture model. Ecotoxicol. Environ. Saf..

[83] Chen Y., Liu Y., Li Y., Yao C., Qu J., Tang J., Chen G., Han Y. (2024). Acute exposure to polystyrene nanoplastics induces unfolded protein response and global protein ubiquitination in lungs of mice. Ecotoxicol. Environ. Saf..

[84] Wu Y., Yao Y., Shen Y., Bai H., Zhang L., Zhang C. (2025). Nanoplastics Chronic Toxicity in Mice: Disturbing the Homeostasis of Tryptophan Metabolism in Gut-Lung-Microbiota Axis. Small.

[85] Wang F., Li Q., Xie C., Zhu N., Deng Y., Li Y., Chen K., Meng W., Wen Y., Liu T. (2025). Dysregulation of SUSD2-CLDN18.2-mediated cell adhesion contributes to lung adenocarcinoma progression associated with chronic low-dose nanoplastics exposure. Ecotoxicol. Environ. Saf..

[86] Amato-Lourenço L.F., Carvalho-Oliveira R., Júnior G.R., Dos Santos Galvão L., Ando R.A., Mauad T. (2021). Presence of airborne microplastics in human lung tissue. J. Hazard. Mater..

[87] Dzierżyński E., Blicharz-Grabias E., Komaniecka I., Panek R., Forma A., Gawlik P.J., Puźniak D., Flieger W., Choma A., Suśniak K. (2025). Post-mortem evidence of microplastic bioaccumulation in human organs: Insights from advanced imaging and spectroscopic analysis. Arch. Toxicol..

[88] Xu H., Dong C., Yu Z., Ozaki Y., Hu Z., Zhang B., Yao W., Yu J., Xie Y. (2024). Detection and analysis of microplastics in tissues and blood of human cervical cancer patients. Environ. Res..

[89] (2012). IARC Working Group on the Evaluation of Carcinogenic Risks to Humans. Arsenic, metals, fibres, and dusts. IARC Monogr. Eval. Carcinog. Risks Hum..

[90] Mossman B.T., Churg A. (1998). Mechanisms in the pathogenesis of asbestosis and silicosis. Am. J. Respir. Crit. Care Med..

[91] Benbrahim-Tallaa L., Baan R.A., Grosse Y., Lauby-Secretan B., El Ghissassi F., Bouvard V., Guha N., Loomis D., Straif K., International Agency for Research on Cancer Monograph Working Group (2012). Carcinogenicity of diesel-engine and gasoline-engine exhausts and some nitroarenes. Lancet Oncol..

[92] Hamra G.B., Guha N., Cohen A., Laden F., Raaschou-Nielsen O., Samet J.M., Vineis P., Forastiere F., Saldiva P., Yorifuji T. (2014). Outdoor particulate matter exposure and lung cancer: A systematic review and meta-analysis. Environ. Health Perspect..

[93] Oberdörster G., Oberdörster E., Oberdörster J. (2005). Nanotoxicology: An emerging discipline evolving from studies of ultrafine particles. Environ. Health Perspect..

[94] Casella C., Cornelli U., Ballaz S., Zanoni G., Merlo G., Ramos-Guerrero L. (2025). Plastic Smell: A Review of the Hidden Threat of Airborne Micro and Nanoplastics to Human Health and the Environment. Toxics.

[95] Sailis A.B. (2026). Could e-cigarette devices generate inhalable micro- and nanoplastics? Exposure plausibility and reproductive relevance. Toxicol. Mech. Methods.

[96] Du Z., Yu X., Li X., Zhang L., Lin Y., He Y., Wu Y. (2025). Mechanism of microplastics in respiratory disease from 2020 to 2024: Visualization and bibliometric analysis. Front. Med..

[97] Islami F., Goding Sauer A., Miller K.D., Siegel R.L., Fedewa S.A., Jacobs E.J., McCullough M.L., Patel A.V., Ma J., Soerjomataram I. (2018). Proportion and number of cancer cases and deaths attributable to potentially modifiable risk factors in the United States. CA Cancer J. Clin..

[98] Wild C.P. (2005). Complementing the genome with an “exposome”: The outstanding challenge of environmental exposure measurement in molecular epidemiology. Cancer Epidemiol. Biomark. Prev..

[99] Brunelli A., Charloux A., Bolliger C.T., Rocco G., Sculier J.P., Varela G., Licker M., Ferguson M.K., Faivre-Finn C., Huber R.M. (2009). European Respiratory Society and European Society of Thoracic Surgeons joint task force on fitness for radical therapy. ERS/ESTS clinical guidelines on fitness for radical therapy in lung cancer patients (surgery and chemo-radiotherapy). Eur. Respir. J..

[100] Ferguson M.K., Durkin A.E. (2002). Preoperative prediction of the risk of pulmonary complications after esophagectomy for cancer. J. Thorac. Cardiovasc. Surg..

[101] Quail D.F., Joyce J.A. (2013). Microenvironmental regulation of tumor progression and metastasis. Nat. Med..

[102] Binnewies M., Roberts E.W., Kersten K., Chan V., Fearon D.F., Merad M., Coussens L.M., Gabrilovich D.I., Ostrand-Rosenberg S., Hedrick C.C. (2018). Understanding the tumor immune microenvironment (TIME) for effective therapy. Nat. Med..

[103] Hirsch F.R., Scagliotti G.V., Mulshine J.L., Kwon R., Curran W.J., Wu Y.L., Paz-Ares L. (2017). Lung cancer: Current therapies and new targeted treatments. Lancet.

[104] Coussens L.M., Werb Z. (2002). Inflammation and cancer. Nature.

[105] Siegfried G., Descarpentrie J., Evrard S., Khatib A.M. (2020). Proprotein convertases: Key players in inflammation-related malignancies and metastasis. Cancer Lett..

[106] Borsig L., Wolf M.J., Roblek M., Lorentzen A., Heikenwalder M. (2014). Inflammatory chemokines and metastasis—Tracing the accessory. Oncogene.

